# High-throughput phenotyping and genetic linkage of cortical bone microstructure in the mouse

**DOI:** 10.1186/s12864-015-1617-y

**Published:** 2015-07-03

**Authors:** Kevin S Mader, Leah Rae Donahue, Ralph Müller, Marco Stampanoni

**Affiliations:** Institute for Biomedical Engineering, University and ETH Zurich, Gloriastrasse 35, Zurich, 8092 Switzerland; Swiss Light Source, Paul Scherrer Institut, WBBA 213, 5352, PSI, Villigen, Switzerland; The Jackson Laboratory, 04609 Bar Harbor, ME USA; Institute for Biomechanics, ETH Zurich, 8093 Zurich, Switzerland

**Keywords:** Phenotyping, Automated 3D imaging, 3D morphology, Quantitative trait loci, Osteocyte lacunae, 3D morphology, Cortical bone, cell shape, Cell distribution, cell alignment

## Abstract

**Background:**

Understanding cellular structure and organization, which plays an important role in biological systems ranging from mechanosensation to neural organization, is a complicated multifactorial problem depending on genetics, environmental factors, and stochastic processes. Isolating these factors necessitates the measurement and sensitive quantification of many samples in a reliable, high-throughput, unbiased manner. In this manuscript we present a pipelined approach using a fully automated framework based on Synchrotron-based X-ray Tomographic Microscopy (SRXTM) for performing a full 3D characterization of millions of substructures.

**Results:**

We demonstrate the framework on a genetic study on the femur bones of in-bred mice. We measured 1300 femurs from a F2 cross experiment in mice without the growth hormone (which can confound many of the smaller structural differences between strains) and characterized more than 50 million osteocyte lacunae (cell-sized hollows in the bone). The results were then correlated with genetic markers in a process called quantitative trait localization (QTL). Our findings provide a mapping between regions of the genome (all 19 autosomes) and observable phenotypes which could explain between 8–40 % of the variance using between 2–10 loci for each trait. This map shows 4 areas of overlap with previous studies looking at bone strength and 3 areas not previously associated with bone.

**Conclusions:**

The mapping of microstructural phenotypes provides a starting point for both structure-function and genetic studies on murine bone structure and the specific loci can be investigated in more detail to identify single gene candidates which can then be translated to human investigations. The flexible infrastructure offers a full spectrum of shape, distribution, and connectivity metrics for cellular networks and can be adapted to a wide variety of materials ranging from plant roots to lung tissue in studies requiring high sample counts and sensitive metrics such as the drug-gene interactions and high-throughput screening.

**Electronic supplementary material:**

The online version of this article (doi:10.1186/s12864-015-1617-y) contains supplementary material, which is available to authorized users.

## Background

In recent years, sequencing genomes has been accelerated manyfold [1, 2]. With a wide availability of reliable genomic information, understanding of complex biological systems is now limited by the ability to develop new and measure subtle changes in phenotypes in an equally rapid rate [3]. In some areas, phenotyping has kept pace through advances in techniques like high-throughput fluorescent [4] and optical computed tomography screening [5]. Both of which allow for hundreds of individuals and phenotypes to be screened in rapid succession and/or in parallel. Particularly in the field of plant genetics, automation in phenotyping has greatly increased the throughput and reliability of genetic studies [6]. However, for high-resolution analyses such as SEM, confocal microscopy, and Synchrotron-based X-ray Tomographic Microscopy (SRXTM), dealing with large numbers of samples (>10) is difficult to impossible for a number of reasons. The first factor is preparation time, since many of these techniques require careful, individual, often human-intensive sample preparation which takes time and scales linearly with the sample count. The second factor is the acquisition time, which on many systems extends above several hours. Consequently entire studies require thousands of hours of pure acquisition, which is tedious and often long enough that imaging characteristics of the involved components can change significantly. Finally once all of the data are collected, the task of extracting meaningful metrics from the images can be even more difficult and time consuming than the initial two tasks. In particular for hierarchical systems with thousands of substructures few standards exist for meaningfully characterizing either the ensemble behavior or the complex relationships between levels in the hierarchy.

Complicating the huge time requirements are the importance of reproducibility, which is impossible when too many human elements are involved. Finally management of all the samples, data, and results, which easily exceed the capacities of analysis tools like Excel (Microsoft,Redmond, USA), R [7], and MATLAB (Mathworks, Natick, USA), make data analysis difficult and time consuming and make data exploration all but impossible.

Even more scalable tools like SQLite are unable to deliver results quickly. Thus, up until now, most large-scale analysis of phenotype on micro- and nanoscale systems has been a gigantic undertaking [8–10]. While much of the work done has provided fascinating insights into the genetic regulation of specific phenotypes, it has lacked a consistent high-throughput reproducible framework that would enable future studies to be easily conducted with minimal time investment.

**Fig. 1:**
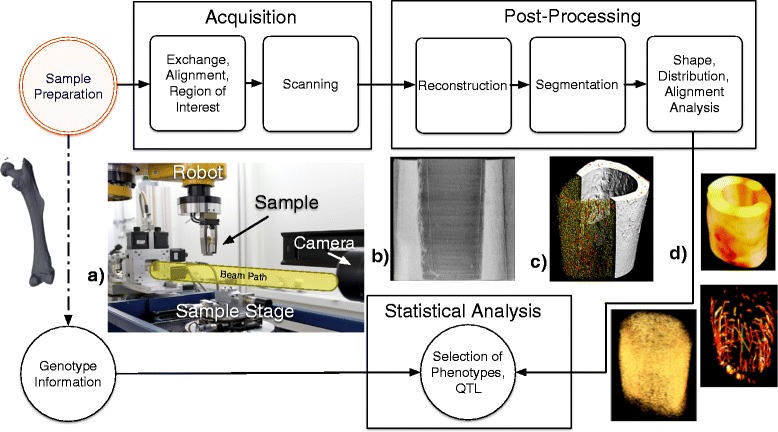
The diagram shows the flow of samples and information from the measurement to the statistical analysis. The automatic exchange system is shown as (**a**). The reconstruction of the x-ray absorption values are shown as (**b**). The segmentation into the different phases is shown as (**c**). Several of the final phenotypes extracted are shown for the cortical bone, canals, and osteocytes in (**d**)

We have developed an optimized, reproducible, automated pathway from sample to final results (Fig. 1). We combine hardware automation involving a robot sample exchange system (Fig. 1a) and software automation for aligning, reconstructing, and analyzing to radically reduce the time investment for performing such a study [11]. Once the images are collected, the analysis is done using an in house post-processing pipeline (Fig. 1b,c) to segment, label, and quantitatively characterize the structures. With new detector technology already enabling scan times below a second and continual improvements in the efficiency and performance of the entire pipeline, the measurements done 2 years ago for this study which took 15 minutes could soon be done in seconds. In light of this streamlining, we propose a new approach to measuring phenotypes and genetic linkage studies which brings the time-scale of these experiments from many years to weeks. We also show how this approach is compatible with many of the new initiatives of open and reproducible science. Using these methods, we identify loci responsible for regulation of cellular-level structure and organization.

We demonstrate the effectiveness of our framework by addressing one of the major challenges of bone biology, understanding bone quality at a cellular scale. Fractures in the femur neck are one of the most debilitating and when surgically treated in the elderly have a mortality rate of 1 in 4 within a year, and require for nearly 1 in 2 cases, additional surgery within 2 years [12]. This bone-strength is multi-factorial, and while bone loss must play an important role, the standard clinical measure of bone mineral density (BMD) only correlates weakly to mechanical properties. Bone quality as defined through structure and organization of bone properties plays a more important role for these cases [13]. Existing studies have examined inheritance in bone microstructure [14–21], but do not use sufficient resolution to characterize the cellular structures inside cortical bone nor manage to disentangle growth hormone during the mapping of specific regions of the genome. To link the microstructure phenotypes to specific gene loci, we performed a large scale study of morphology and cortical micro-structure on a population of mice raised in a controlled environment with known genetic lineage, an intercross-experiment. The mice were 2nd generation offspring from two different commonly used strains of mice with high (C3H/HeJ) and low (C57BL/6J) bone mass with both strains homozygous for a mutation in the growth hormone releasing hormone receptor. We then measured and statistically analyzed these results with the tools of quantitative trait localization to establish explanatory models for phenotype variation and produce a map enabling the targeted search for specific genes involved in bone quality. This will lead to the elucidation of potential mechanisms of regulation and maintenance of bone strength. Since the homology between murine and human genomes is over 75 % [22, 23], we hope to make inroads towards the targeted identification of high-risk patients based on genetic screening, and a personal medicine-based approach to treatment.

## Results

**Fig. 2:**
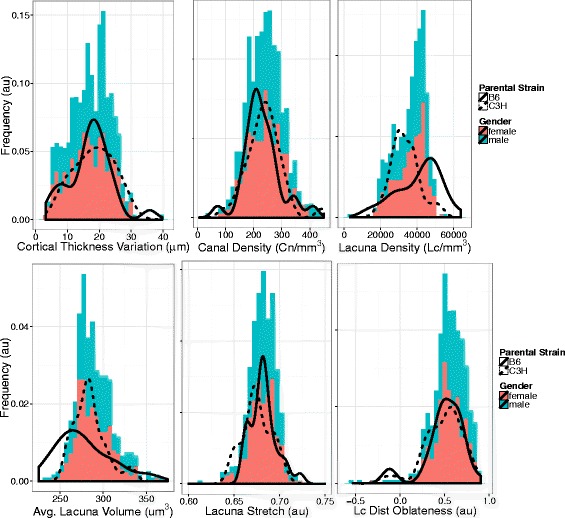
The distribution and heritability for the selected microstructural metrics for QTL analysis: cortical thickness variation, canal density, lacuna density, lacuna volume, lacuna stretch, lacuna distribution oblateness. The values are shown as histograms (x-axis is metric value and y-axis is relative animal count) with female colored in pink and male colored in blue. The distributions for the respective parental strains are shown as the black curves with different line styles indicating the parental strain

**Fig. 3:**
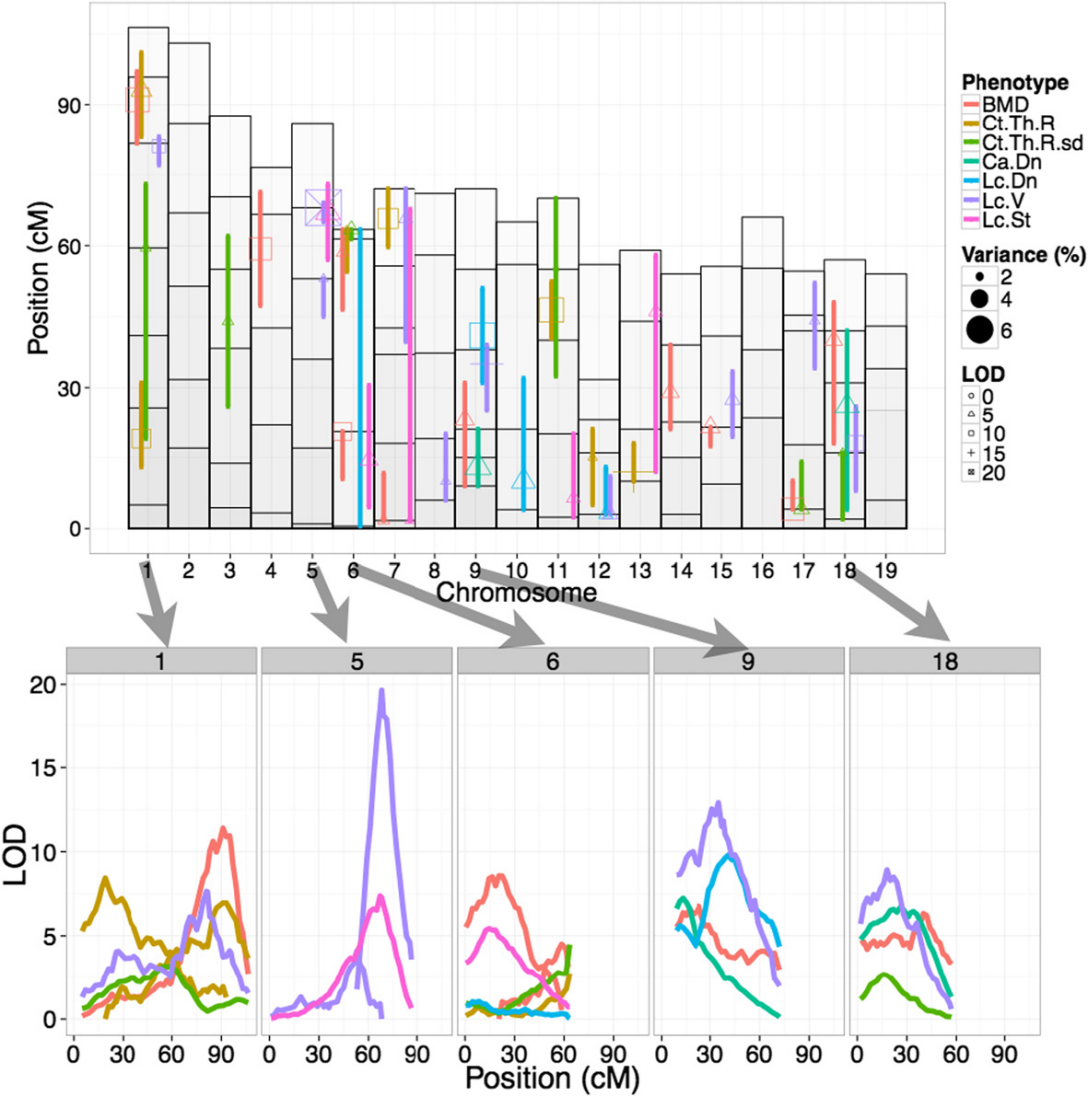
The fitted QTL model for every phenotype where a model could be found according to the procedure and thresholds as described in the methods section (no model was found for Lacuna Distribution Oblateness). The top graph shows the 19 autosomes (the sex chromosomes are not examined in this study) along the X-axis and the position on the chromosome in centimorgans on the Y-axis. The markers tagged are shown as white boxes with black borders. Every phenotype is shown in a different color and each loci is shown by a symbol indicating the LOD score of the corresponding QTL. The size of the symbol indicates how much of the population variance is explained by that QTL. The colored line represents the 95 % confidence region for the location of the loci. For selected chromosomes with high degrees of overlap (1,5,6,9,18) the LOD scores from the 0.1cM refinement are shown in the bottom figures to show the similarities between the LOD-score and thus providing indications of pleiotropy at these loci

**Fig. 4:**
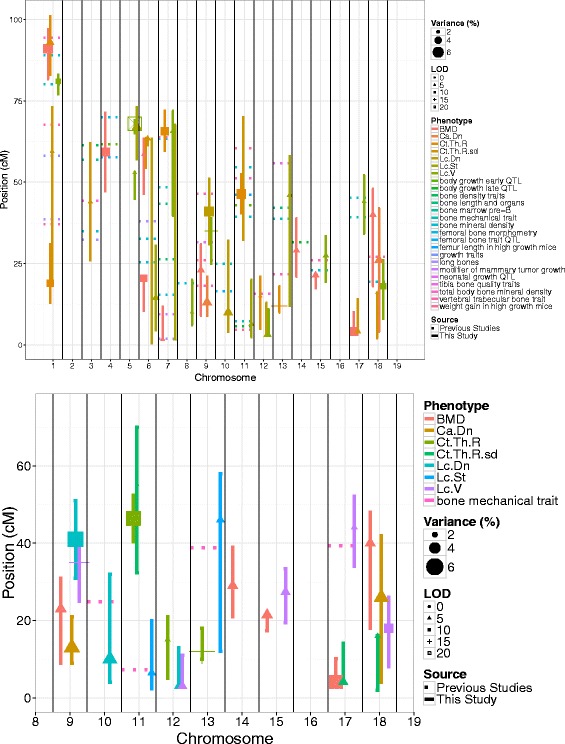
The graphs show the QTL calculated in this study (solid line) against the QTL calculated in previous studies (dotted lines). The x-axis indicates the chromosome and the y-axis shows the position on the chromosome in centimorgans. Different colored lines and points designate the different phenotypes. The shape and size of the point shows the LOD score and contributed variance (%) respectively. The length of the line indicates the 95 % confidence interval as determined from the LOD curves. The top graph shows all overlapping QTL related to bone and growth while the bottom shows just the ones relevant for mechanical properties

We found that the heritability criteria (>40 %) were met for the following metrics: Cortical Thickness Variation (Ct.Th.R.sd), Canal Density (Ca.Dn), Lacuna Density (Lc.Dn), Mean Lacuna Volume, Lacuna Stretch (Lc.St), and Lacuna Distribution Oblateness (Lc.Dt.Ob) described in [24] (Fig. 2). The distribution of additional phenotypes in the F2 group is shown in the Additional file 1. We then determined the threshold or penalty criteria (shown in Additional file 1: Table S1) for significance and interaction for each of the phenotypes through the permutation approach discussed in the methods. These values were used to fit QTL models for the selected phenotypes plus Bone Mineral Density and Cortical Thickness for comparison. The QTL models were found for every phenotype except for Distribution Oblateness. The models that were found were able to explain 8–40 % of the F2 population variance using between 2 and 10 loci. The models and LOD curves for several particularly loci-rich chromosomes are shown in Fig. 3. From these models, we found the markers within the 95 % confidence interval as calculated using the LOD curves, which had previously been linked to other phenotypes. We then selected several metrics potentially specific for the microstructural parameters we investigated (Fig. 4). The loci and respective markers are shown in Additional file 1: Table S5.

**Overlapping markers** The table and graphs shown in Table 1 and Figs. 3 and 4 demonstrate the overlap between the results of this study and a wide variety of published loci. Since our initial intent was to better understand bone quality in the context of mechanical properties, we will focus most intently on the overlap with mechanically-relevant markers, which are shown in Table 2. The most interesting results are the loci on Chromosome (Chr)10 and Chr11 since they are independent of BMD (as indicated by the shading). The corresponding phenotypes from this study are lacuna number density (Lc.Dn) and lacuna stretch (Lc.St). The other relevant traits at these loci are different from ours, and the first is related to femoral bone, while the second to vertebral bone and body growth.

**Table 1:** QTL Models (phenotypes and explained variance). The table shows the positions for the identified loci (rows) for each phenotype (columns). For each loci the additive component is shown as being either C3H >B6 or C3H <B6 indicating in which genotype the phenotype value is higher. Dominance, if present, is shown by a (D) next to the appropriate parental strain. Finally the variance explained and LOD score (in parentheses) are shown as determined by removing that term from the analysis. Although several of the models involved interactions they are not indicated in this table

Chr	Pos (cM)	BMD	Cortical thickness	Cortical thickness variance	Canal density	Lacuna density	Mean lacuna volume	Mean lacuna stretch
1	89.9	C3H (D) >B6, 5.73 (12.77)						
1	22.3		C3H >B6, 4.07 (8.53)					
1	97.1		C3H >B6, 3.42 (7.20)					
1	59.0			C3H >B6, 1.71 (3.13)				
1	81.5						C3H >B6, 2.28 (6.16)	
3	39.2			C3H <B6, 1.81 (3.32)				
4	52.1	C3H >B6, 4.82 (10.81)						
5	53.8						C3H >B6, 1.68 (4.55)	
5	68.0						C3H >B6 (D), 6.97 (18.11)	
5	68.2							C3H >B6 (D), 3.45 (6.52)
6	20.8	C3H <B6, 3.33 (7.55)						
6	61.7	C3H (D) >B6, 2.10 (4.80)						
6	63.4		C3H >B6, 1.08 (2.31)	C3H >B6, 2.35 (4.29)				
6	4.1					C3H (D) <B6, 0.53 (1.00)		
6	18.9							C3H <B6 (D), 2.77 (5.25)
7	2.2	C3H >B6, 1.91 (4.38)						
7	60.6		C3H >B6, 4.24 (8.89)					
7	43.3						C3H >B6, 2.60 (7.00)	
7	1.7							C3H (D) <B6, 1.46 (2.80)
8	18.2						C3H (D) <B6, 1.85 (5.02)	
9	12.0	C3H <B6, 3.32 (7.51)						
9	13.2				C3H (D) <B6, 4.15 (7.25)			
9	40.1					C3H <B6, 5.59 (10.26)		
9	32.2						C3H <B6, 4.86 (12.85)	
10	17.8					C3H >B6, 3.88 (7.19)		
11	45.6		C3H >B6, 4.53 (9.47)					
11	54.1			C3H >B6 (D), 1.27 (2.34)				
11	8.8							C3H >B6, 2.38 (4.52)
12	16.2		C3H (D) <B6, 1.79 (3.81)					
12	3.0					C3H <B6, 2.19 (4.09)		
12	3.6						C3H <B6, 3.44 (9.20)	
13	10.3		C3H >B6 (D), 5.81 (12.05)					
13	46.2							C3H >B6, 2.25 (4.28)
14	22.1	C3H >B6, 2.17 (4.96)						
15	21.5	C3H (D) >B6, 2.92 (6.63)						
15	27.7						C3H >B6, 2.36 (6.35)	
17	4.9	C3H <B6, 4.44 (9.99)						
17	7.9			C3H >B6 (D), 2.65 (4.83)				
17	45.3						C3H <B6, 1.74 (4.72)	
18	44.5	C3H >B6, 2.80 (6.36)						
18	15.4			C3H (D) <B6, 1.64 (3.00)				
18	23.5				C3H >B6, 3.84 (6.72)			
18	14.6						C3H >B6, 3.16 (8.46)

**Table 2:** A list of QTL from this study which have overlap with previously determined mechanical QTL. The first five columns show the chromsome, peak position, and starting and ending positions of the 95 % confidence interval for each QTL as determined in this study. The fifth column shows the overlapping QTL from the QTLArchive. The shaded rows indicate QTL which do not overlap at all with BMD and are of particular interest for further investigation

Chr.	Pos.(cM)	Start	End	Phen.	Overlapping QTL (MGI Accession ID)
**10**	**10**	**4**	**32**	**Lc.Dn**	**bone mechanical trait (MGI:3511309), femoral bone trait QTL (MGI:3701623)**
**11**	**6**	**2**	**20**	**Lc.St**	**bone mechanical trait (MGI:3511310), vertebral trabecular bone trait (MGI:3045053), body growth late QTL (MGI:108503)**
13	46	12	58	Lc.St	bone mechanical trait (MGI:3511311), bone mineral density (MGI:2389099), bone length and organs (MGI:3639971), femoral bone trait QTL (MGI:3701625), tibia bone quality traits (MGI:3721636), vertebral trabecular bone trait (MGI:3045057), body growth late QTL (MGI:108493)
17	44	34	52	Lc.V	bone mechanical trait (MGI:3511312), bone mineral density (MGI:3694913)

The second focus was to understand properties which are independent of BMD, since BMD has been so well characterized in mice and human populations and is easily measurable. These results are summarized in Table 3. Curiously one of the BMD loci we found has not previously been identified and thus might be of interest for further investigation to determine the validity of this QTL.

**Table 3:** A list of QTL from this study which have no overlap with previously determined BMD QTL. The first five columns show the chromsome, peak position, and starting and ending positions of the 95 % confidence interval for each QTL as determined in this study. The fifth column shows the overlapping QTL from the QTLArchive. The shaded rows indicate QTL which are also mechanically relevant

Chr.	Pos.(cM)	Start	End	Phen.	Overlapping QTL (MGI Accession ID)
4	59	47	71	BMD	femoral bone trait QTL (MGI:3701621), femoral bone morphometry (MGI:2154763), body growth early QTL (MGI:108558)
7	2	2	12	BMD	long bones (MGI:3639954), modifier of mammary tumor growth (MGI:1890552)
7	66	60	72	Ct.Th.R	femoral bone morphometry (MGI:2154761), vertebral trabecular bone trait (MGI:3045046)
9	13	9	21	Ca.Dn	vertebral trabecular bone trait (MGI:3045048)
9	23	9	31	BMD	vertebral trabecular bone trait (MGI:3045048), vertebral trabecular bone trait (MGI:3045049)
9	35	25	39	Lc.V	vertebral trabecular bone trait (MGI:3045049), neonatal growth QTL (MGI:3640530)
**10**	**10**	**4**	**32**	**Lc.Dn**	**bone mechanical trait (MGI:3511309), femoral bone trait QTL (MGI:3701623)**
**11**	**6**	**2**	**20**	**Lc.St**	**bone mechanical trait (MGI:3511310), vertebral trabecular bone trait (MGI:3045053), body growth late QTL (MGI:108503)**
12	15	5	21	Ct.Th.R	tibia bone quality traits (MGI:3721634)
14	29	21	39	BMD	body growth early QTL (MGI:108480), body growth late QTL (MGI:1932377)

**Comparison with previous work** The two QTL of highest interest, as mentioned in previous section, are the loci on Chr10 at 10cM and Chr11 at 6cM. The mechanically relevant genes were in both cases from the same work done by Volkmann et al. [25]. In their work, the mice were not the same as in our study and were instead a cross between C3H/HeJ and DBA/2J, with no known mutation in *Ghrhr*. The tag located closest to the Volkmann QTL, D10Mit40 (24.87cM), was located on Chr10 at position 29cM. It was associated with both Ultimate and Post Yield displacement of the femur independent of femur geometry and body weight. The second QTL reported by Volkmann overlapped with the tags D11Mit2 and D11Mit16 located on Chr11 at position 7.3 and 2.94cM respectively.

These tags, while not independent of femur geometry, were associated with predicted modulus, yield load, and stiffness. The QTL located in this study on Chr5 are also of significant interest because they have high LOD scores and do not overlap with previously identified phenotypes possibly indicating a new gene involved only in the microstructure of bone. It is important to reiterate that these QTL are the ones, which have been published in the QTL Archive run by the Churchill Group at the Jackson Laboratory (http://www.qtlarchive.org/) and are thus not inherently exhaustive.

## Discussion

The experiments and analysis we have done show the utilityand wide-spread potential of automated synchrotron-based tomographic microscopy and the associated post-processing pipeline for phenotyping large, complicated morphological phenotypes. In bone particularly, the microstructure can be well resolved and quantitatively characterized in cell shape, distribution, and alignment over millions of structures. The ability to link these phenotypes with genetic background and consequently other macroscopic, mechanical, and endocrine phenotypes provides a more complete picture of how the microstructure is correlated to existing, well-understood characteristics.

The genetic linkage results confirm loci found by earlier experiments. The mechanically relevant loci found provide the first hints at what microstructural properties might explain the variations in deformation under mechanical load. The results also shed light on several new loci, which might regulate bone structure using previously unidentified mechanisms. Since much is still not known about the modeling and remodeling of bone at the cellular level, these genes might make some of these easier to understand. If we assume the approximate density of genes in the mouse genome is 16 per cM [26], an interval of 0.0625 should be used in order to obtain single gene resolution. Due to the sparsity of the identified markers no significant improvement was seen when intervals of less than 0.1cM were used for scanning the genome. Thus the current dataset is not sufficient for identifying single genes and future studies will need to be done before specific genes can be analyzed. While it is too early to identify specific genetic homologues, a comparison of the identified QTL and human-mouse homologous genes shows many matches and trends where large portions of the mouse genome are consistently mapped to the same chromosome of the human genome indicating a high degree of conservation between the two strains(see Additional file 1). This conservation indicates great promise for finding relevant human genes in future studies. The work done in the study lays groundwork for both deeper investigation of murine biomechanics and future possibilities in high-throughput genome-scale imaging. Both of these tasks require fast, predictable, quantitative tools to investigate structure and function. The results primarily serve as the first map of the microstructural genome space in mice without GH. While the resolution we obtained is far from the single gene level, it allows past and future studies to compare the results with these quantities, which are traditionally difficult to measure. Additionally our results provide support to conduct further congenic and eventually knock-out studies to investigate and better localize the genes involved in these metrics. In particular the D10Mit40, D11Mit2, D11Mit16 markers should be carefully examined. In combination with mechanical testing congenic strains of these mice could reveal the connection between osteocyte lacunar density and shape and the mechanical properties of bone. Further investigations could also provide insight into how morphometric metrics can be interpreted in standard samples. The lack of GH in these mice removes a significant confounding factor when identifying the unique QTL for specific phenotypes. However, for applicability in further studies, the effect of GH must be included. In Mader et al. [24] a comparison was made between two strains from the same parental lines as those in this study, particularly looking at mice with and without *Ghrhr*. The results showed many differences between mice with and without GH. Particularly with metrics like Mean Lacuna Volume (and correspondingly lacuna dimensions) and spatial distribution, GH plays a significant role and makes inferring the specific differences caused by non-growth-hormone genetic background difficult. This study serves as a proof of concept for the capabilities of high-throughput tomographic X-ray microscopy in combination with a streamlined post-processing and analysis pipeline. While this study only investigated morphological and structural phenotypes of bones, given the high sensitivity of phase contrast to biological materials and the availability of contrast agents, studies investigating more complicated biologically relevant phenotypes are conceivable in the immediate future. In particular studying the interactions between environmental stimulus and different genetic backgrounds could be of high interest and such studies necessitate very large sample sizes.

## Conclusions

Beyond biological applications, the framework is applicable to a wide range of high-throughput applications where large sample counts are required to achieve statistical significance. In material science, large numbers of samples measured consistently with detailed quantitative information are required to establish a link between process, structure, and mechanical behavior. Based on our experience with the hundreds of imaging users at the Swiss Light Source, we have noticed a trend in the distribution of time spent preparing experiments and analyzing the results. Much as the genetics community has experienced several years ago, computed tomography measurements and experiments have become manyfold faster and easier. This has shifted much more of the burden on the analysis, with the distribution of work, in many cases being thousands of times longer for the analysis than the experiments themselves.

The developments we have made can drastically rebalance the time distribution of scientists examining the structures of complex materials, away from monotonous, repetitive, operator-biased modalities of image processing towards interpretation of vast, rich quantitative results. We think the quality and agility of research can be drastically improved. A faster post-processing pipeline means that study groups and targets can be screened much more easily and a feedback loop can be established so that studies can be tuned in nearly real-time for promising and poor results.

## Methods

To ascertain the genetic contribution to murine femur cortical microstructure we performed a standard intercross experiment. The two background strains used, C3H/HeJ (C3H) and C57BL/6J (B6), were selected because of their previously shown [27] strong variations in cortical structure. Both of the parental strains - C3.B6-*Ghrhr*^*lit*^/J and C57BL/6J-*Ghrhr*^*lit*^/J - were homozygous for a spontaneous mutation in the growth hormone releasing hormone receptor (*Ghrhr*) causing undetectably low circulating growth hormone (GH) levels [16]. The mice were raised and sacrificed at the Jackson Laboratory. Animalstudies were approved by the Institutional AnimalCare and Use Committee of The Jackson Laboratory(Bar Harbor, ME, USA) and performed according to Swiss law. We examined 12 of the F1 and 1132 of the F2 population comprised of both male and female mice with a 47 % male and 53 % female split. The F1 population size was established by the number of samples available, and, due to their identical genetic background, their primary function is in assessing the environmental variance. The F2 population size was determined by examining the micro scale phenotypes measured in [16] and determining the number of samples required such that the baseline for variation, as determined through the permutation test, left multiple loci for the traits which were statistically determined to have multiple loci.

### Measurements

The femurs were extracted from four month-old mice and were measured locally in the mid-diaphysis at high-resolution (1.4 *μ*m voxel size) using synchrotron-based tomographic microscopy at TOMCAT using automatic sample exchange, alignment, and region of interest identification [11] with a field of view 1.5 mm x 1.5 mm x 1.5 mm. Standard procedures were used to reconstruct, segment, and morphologically analyze the data [11, 16, 24, 27].

### Morphometric analysis

We introduce two additional possible phenotypes for this investigation for the purpose of better characterizing cortical bone distribution: radial cortical thickness (Ct.Th.R) and radial cortical thickness standard deviation (Ct.Th.R.sd). The radial cortical thickness is calculated using the standard procedure [28], but where the average (first metric) and standard deviation (second metric) are not taken as a volume average but rather as the average thickness when examining the shell in polar coordinates with the center of the bone aligned with the Z-axis. Since both of these methods are weighted by angle rather than volume, the result is less biased by thicker regions, which naturally have high volumes. We developed the initial experiment to study all micro structural metrics [24] applied to the cortical shell, osteocyte lacunae, and canal structures independent of heritability. The entire list of metrics and the single scans are included in the Additional file 1.

While a full description for calculating these metrics from a 3D dataset is available in [24], we provide in this manuscript basic explanations of the most critical metrics. The number density metrics (Ca.Dn, Lc.Dn) are calculated by determining the number of these distinct objects (canals and lacunae respectively) which can be found in 1 *mm*^3^ of calcified bone tissue. Mean Lacuna Volume (Lc.V) is model-free and is calculated by counting the total number of empty voxels inside the given lacuna object. Finally Lacuna Stretch (Lc.St) indicates the anisotropy of the lacuna with a number between 0 (completely spherical) to 1 (completely rod or plate-like). Finally Lacuna Distribution Oblateness indicates the type of anisotropy present in the spatial distribution in the spatial distribution: prolate (indicating that the lacunae are spaced closely in two orthogonal directions and further apart in the remaining or oblate where the lacunae are spared far apart in two orthogonal directions and close in only one.

### QTL Mapping

From these metrics, we selected a subset to pursue further with QTL analysis based on a broad-sense heritability criterion of 40 %. The heritability was estimated by comparing the variation between the F1 and F2 generations. The environmental variance was estimated using the metric variance measured in the F1 population, which being genetically identical show only the variation due to environmental factors, genetic drift, and measurement error, which together provide an estimate for the uncertainty in the examined systems. The genetic lineage of the F2 mice was identified by using polymerase chain reaction (PCR) markers of the at 98 locations distributed among the 19 autosomes. The markers used were MIT, a type of microsatellite marker, that consists of a short sequence with multiple tandem repeats enabling them to be utilized well on these closely related strains. The parental strain for each given allele can be determined by comparing them to genomic DNA. Genotype information was only available for 755 of the 1132 mice; thus for the rest of the study only these 755 will be considered.

The genotype probabilities were estimated at intervalsof 2cM and 128 draws were taken to better estimate the actual probabilities at these inter-marker locations. The first activity level for various regions of the genome was estimated by performing a standard single-locus genome scan [29] for each phenotype. We used the extended Haley-Knott method for performing the single-locus scans because of its robustness and utilization of genotype information [30]. We accounted for gender discrepancies between the strains by including gender as covariant to the model. The covariant is introduced as a so-called interactive covariant where it can have both an additive effect and interact with the genotype [29]. Genome-wide thresholds for significance and interaction were selected by performing a 2 loci scan again over 1000 permutations of the phenotype over the genotypes (and are shown in the Additional file 1). As standard in QTL studies, the test used for statistical significance is the logarithm of the odds (LOD) score, a logarithmic score indicating the probability over the null hypothesis (no QTL). A LOD score of 3 refers to 1:1000 [31]. The full QTL models for each phenotype were created following the automated forward/backward method described in [30]. The thresholds and penalties for main effect, heavy and light interaction were determined genome-wide for each phenotype by performing 1000 permutations of the two loci scan and using a 5 % significance level. The contribution of each loci to the final variance was computed by comparing models with and without the given term. The LOD plots for each model were created during this refinement stage with 0.1cM step size to best show the positions and shape of the loci (Fig. 3).

### Statistical analysis and implementation

The analysis was performed using R (3.0.1) [7] using R/QTL [32] to run the analysis. To generate the visualizations, we used ggplot2 in combination with plyr [33, 34]. The data for comparing to older studies was acquired from QTLArchive.org and processed using an R script. This along with the tools to generate all of the plots are available at: http://dx.doi.org/10.6084/m9.figshare.726136.

## Additional file

Additional file 1
**Supplemental materials.**
