# Supplementary material for: High-throughput phenotyping and genetic linkage of cortical bone microstructure in the mouse
Source: BMC Genomics. 2015 Jul 3;16(1):493. doi: 10.1186/s12864-015-1617-y (PMC4490749; doi:10.1186/s12864-015-1617-y)
Supplement: Additional file 1 — Supplemental materials. [file 12864_2015_1617_MOESM1_ESM.pdf]

Supplemental Materials

Pipeline Analysis

The details of the measurements and phenotypes are described in detail in [24, 15]. For this study, however, both techniques had to be significantly upscaled to handle the 1300 different samples which were measured. The basic script used to calculate the structural phenotypes from the image data is available at https://gist.github.com/kmader/3980f2b1768af4c1e1d2. It has dependencies on our local, in house frameworks, but it covers the flow and processing steps required for the final analysis.

Hardware Infrastructure

The analysis was run on a local High Performance Computing (HPC) cluster called Merlin4 and maintained by the IT department at the Paul Scherrer Institut in Villigen, Switzerland. The cluster consisted of 30 machines each with 12 cores and 2-4 GB of memory per core.

Software Infrastructure

Given the large number of samples, and need to share the resources, the samples were run individually using Sun Grid Engine (Oracle Corporation). The script used to run the jobs is also available at https://gist.github.com/kmader/3980f2b1768af4c1e1d2#file-ufem-sge. Since the analysis was time consuming (¿10 hours per sample), the analysis was divided into smaller parts (4 roughly equal in duration) so they could be scheduled easier.

Storage and Analysis

Given the large amount of meta-data (cell and canal information) produced by the analysis, examining and interpreting these results required the use of a database. To this end we setup a MySQL Server (Oracle Corporation) which stored all of the results and could be queried easily from R, Python, and Matlab.

Complete Phenotype List

The complete set of phenotypes measured used along with their descriptions and a plot of their LOD values (figure S1) using the single locus scan method described in the Methods section.

- BMD - Bone Mineral Density
- Cn.Align - Canal Alignment (Anisotropy of Alignment Tensor from Primary Canal Orientations)
- Cn.D - Canal Number Density (#/mm^3)
- Cn.N - Canal Count (#)
- Cn.V - Average Canal Volume (μm^3)
- Cn.Vert - Canal Verticalness (Average Projection of Primary Canal Orientation along the Z axis)
- Cn.Vf (%) - Canal Volume Fraction (Total Volume of Canals / Total Volume of Calcified Bone)
- Ct.Th - Average Cortical Thickness (by voxel) in mm
- Ct.Th.sd - Standard Deviation of Cortical Thickness (by voxel) in mm

- Ct.Th.R - Average Cortical Thickness (by radial angle) in voxels
- Ct.Th.R.sd - Standard Deviation of Cortical Thickness (by radial angle) in voxels
- Lc.Align - Primary Lacunar Alignment (Anisotropy of Alignment Tensor from Primary Lacunae Orientations)
- Lc.Align2 - Secondary Lacunar Alignment (Anisotropy of Alignment Tensor from Secondary Lacunae Orientations)
- Lc.D - Lacuna Number Density (#/mm^3)
- Lc.Dt.Ob - Lacunar Distribution Oblateness (Oblateness of the Distribution Tensor for Lacuna)
- Lc.Dt.St - Lacunar Distribution Stretch (Stretch/Anisotropy of the Distribution Tensor for Lacuna)
- Lc.N - Lacuna Count (#)
- Lc.Ob - Average Lacunar Oblateness
- Lc.St - Average Lacunar Stretch
- Lc.V - Average Lacunar Volume (μm^3)
- Lc.Vert - Lacuna Verticalness (Average Projection of Primary Lacuna Orientation along the Z axis)
- Lc.Vf (%) - Lacunar Volume Fraction (Total Volume of Lacunae / Total Volume of Calcified Bone)

While these metrics characterize the given sample fairly well they poorly account for the inhomogeneity inside a sample. This inhomogeneity could be quite important for bone strength particularly when considering fracture events, since they might create weak regions more susceptible or possibly points of invitation for fracture. Future studies should investigate metrics to assess this spatial inhomogeneity.

Correlation between Phenotypes

In figure S3, we show the correlation between the selected phenotypes.

Differences between Male and Female

Since gender was used as a covariate in the analysis the differences between the male and female mice in the F2 population are shown in table S1. The LOD-score plot was also generated individually for male and female (figure S4) and the result shows minimal difference between the genetic dependence of specific phenotypes.

Table S1: Comparison between male and female mice of the F2 generation. The p-value indicates the differences are significant. The units are given in the previous section.

	female N=414	male N=341	p.overall
BMD	0.03 (0.00)	0.03 (0.00)	0.32
Ct.Th	0.18 (0.01)	0.18 (0.02)	0.57
Ct.Th.sd	0.02 (0.00)	0.02 (0.00)	0.73
Ct.Th.R	82.3 (6.89)	80.7 (7.58)	<0.01

continued on next page

Table S1 – *continued from previous page*

	female<br>N=414	male<br>N=341	p.overall
Ct.Th.R.sd	16.2 (5.36)	16.4 (5.96)	0.51
Cn.V	29906 (15181)	35262 (22998)	<0.01
Cn.N	194 (70.0)	191 (65.2)	0.47
Cn.D	240 (70.3)	237 (67.0)	0.57
Cn.Vf (%)	0.01 (0.00)	0.01 (0.01)	<0.01
Cn.Align	0.77 (0.07)	0.79 (0.06)	<0.01
Cn.Vert	47.9 (4.12)	49.3 (3.69)	<0.01
Lc.N	29319 (6793)	30958 (7220)	<0.01
Lc.V	284 (20.5)	289 (23.5)	<0.01
Lc.D	36803 (7737)	38833 (7911)	<0.01
Lc.Vf (%)	0.01 (0.00)	0.01 (0.00)	<0.01
Lc.St	0.69 (0.01)	0.68 (0.01)	<0.01
Lc.Ob	-0.34 (0.04)	-0.34 (0.05)	0.24
Lc.Align	0.86 (0.03)	0.85 (0.04)	<0.01
Lc.Vert	1.00 (0.00)	0.99 (0.03)	0.13
Lc.Align2	0.55 (0.08)	0.54 (0.08)	0.08
Lc.Dt.St	0.29 (0.13)	0.33 (0.15)	<0.01
Lc.Dt.Ob	0.53 (0.16)	0.53 (0.20)	0.71

Differences at Highlighted QTLs

Here we show the differences at the highlight QTLs for their mechanical importance and lack of BMD overlap. The first is on chromosome 10 at apposition of 10cM shown in table S2.

Table S2: Comparison between all phenotypes in mice with various genotypes at marker D10Mit77 at position 4cM on chromosome 10. The units are given in the previous section. The p-values were calculated by comparing the 3 groups (2 homozygous, 1 heterozygous) and the trending p-value is checking specifically for additive inheritance.

	B6/B6<br>N=189	B6/C3H<br>N=357	C3H/C3H<br>N=172	p.overall	p.trend
BMD	0.03 (0.00)	0.03 (0.00)	0.03 (0.00)	0.62	0.49
Ct.Th	0.18 (0.01)	0.18 (0.01)	0.18 (0.01)	<0.01	<0.01
Ct.Th.sd	0.02 (0.00)	0.02 (0.00)	0.02 (0.00)	0.01	0.03
Ct.Th.R	82.7 (6.90)	81.5 (7.39)	80.2 (7.33)	0.01	<0.01
Ct.Th.R.sd	17.1 (5.30)	16.0 (5.77)	16.0 (5.57)	0.08	0.05
Cn.V	31555 (20537)	32519 (19545)	32334 (17309)	0.85	0.69
Cn.N	188 (72.8)	191 (68.0)	201 (63.6)	0.16	0.08
Cn.D	231 (73.8)	236 (69.2)	252 (63.0)	0.01	<0.01
Cn.Vf (%)	0.01 (0.01)	0.01 (0.00)	0.01 (0.00)	0.37	0.16
Cn.Align	0.77 (0.07)	0.78 (0.07)	0.77 (0.06)	0.16	0.68
Cn.Vert	48.1 (3.78)	48.8 (4.13)	48.7 (3.59)	0.13	0.14
Lc.N	28799 (7404)	30303 (6813)	31111 (6938)	0.01	<0.01
Lc.V	286 (22.2)	286 (21.8)	287 (22.7)	0.92	0.88

continued on next page

Table S2 – *continued from previous page*

	B6/B6 N=189	B6/C3H N=357	C3H/C3H N=172	p.overall	p.trend
Lc.D	35758 (8196)	37973 (7502)	39740 (7964)	<0.01	<0.01
Lc.Vf (%)	0.01 (0.00)	0.01 (0.00)	0.01 (0.00)	<0.01	<0.01
Lc.St	0.69 (0.01)	0.69 (0.01)	0.68 (0.01)	0.01	<0.01
Lc.Ob	-0.33 (0.04)	-0.34 (0.04)	-0.34 (0.04)	0.19	0.13
Lc.Align	0.85 (0.04)	0.86 (0.03)	0.85 (0.03)	0.18	0.20
Lc.Vert	1.00 (0.00)	1.00 (0.00)	0.99 (0.04)	0.13	0.09
Lc.Align2	0.54 (0.09)	0.55 (0.08)	0.56 (0.08)	0.06	0.02
Lc.Dt.St	0.28 (0.14)	0.31 (0.14)	0.33 (0.15)	<0.01	<0.01
Lc.Dt.Ob	0.51 (0.19)	0.54 (0.17)	0.55 (0.17)	0.03	0.01

The second is on chromosome 11 at a position of 6cM shown in table S3

Table S3: Comparison between all phenotypes in mice with various genotypes at marker D11Mit2 at position 2.4cM on chromosome 11. The units are given in the previous section. The p-values were calculated by comparing the 3 groups (2 homozygous, 1 heterozygous) and the trending p-value is checking specifically for additive inheritance.

	B6/B6 N=165	B6/C3H N=264	C3H/C3H N=160	p.overall	p.trend
BMD	0.03 (0.00)	0.03 (0.00)	0.03 (0.00)	0.34	0.24
Ct.Th	0.18 (0.01)	0.18 (0.01)	0.18 (0.01)	<0.01	<0.01
Ct.Th.sd	0.02 (0.00)	0.02 (0.00)	0.02 (0.00)	0.53	0.39
Ct.Th.R	79.9 (7.55)	81.6 (7.40)	82.6 (7.12)	<0.01	<0.01
Ct.Th.R.sd	16.3 (5.78)	16.2 (5.51)	16.3 (5.83)	0.99	1.00
Cn.V	32500 (22090)	31251 (16581)	30656 (19779)	0.67	0.38
Cn.N	180 (68.6)	200 (66.5)	186 (65.0)	<0.01	0.35
Cn.D	230 (71.0)	248 (69.2)	232 (68.1)	0.01	0.75
Cn.Vf (%)	0.01 (0.01)	0.01 (0.00)	0.01 (0.00)	0.49	0.41
Cn.Align	0.78 (0.06)	0.78 (0.07)	0.77 (0.06)	0.46	0.24
Cn.Vert	48.9 (3.89)	48.6 (4.05)	48.4 (3.66)	0.50	0.24
Lc.N	29163 (7113)	30550 (7103)	30058 (7252)	0.15	0.25
Lc.V	283 (22.4)	288 (22.9)	289 (23.6)	0.04	0.02
Lc.D	37806 (8105)	38345 (7995)	37306 (8337)	0.43	0.59
Lc.Vf (%)	0.01 (0.00)	0.01 (0.00)	0.01 (0.00)	0.40	0.81
Lc.St	0.68 (0.01)	0.69 (0.01)	0.69 (0.01)	<0.01	<0.01
Lc.Ob	-0.34 (0.04)	-0.34 (0.05)	-0.33 (0.05)	0.22	0.15
Lc.Align	0.85 (0.04)	0.85 (0.03)	0.86 (0.03)	0.07	0.03
Lc.Vert	1.00 (0.01)	1.00 (0.03)	1.00 (0.00)	0.62	0.65
Lc.Align2	0.54 (0.09)	0.55 (0.08)	0.56 (0.09)	0.32	0.13
Lc.Dt.St	0.30 (0.14)	0.32 (0.15)	0.31 (0.15)	0.36	0.31
Lc.Dt.Ob	0.53 (0.18)	0.52 (0.19)	0.55 (0.16)	0.46	0.31

Interacting QTLs

Since interaction between loci was allowed, we show an example (fig. S5) where the value and behavior of a locus changes based on the genotype at another locus. The values in the graph illustrate some of the complexity in the results and demonstrate the importance of including such interaction terms in the analysis (which increases computational time).

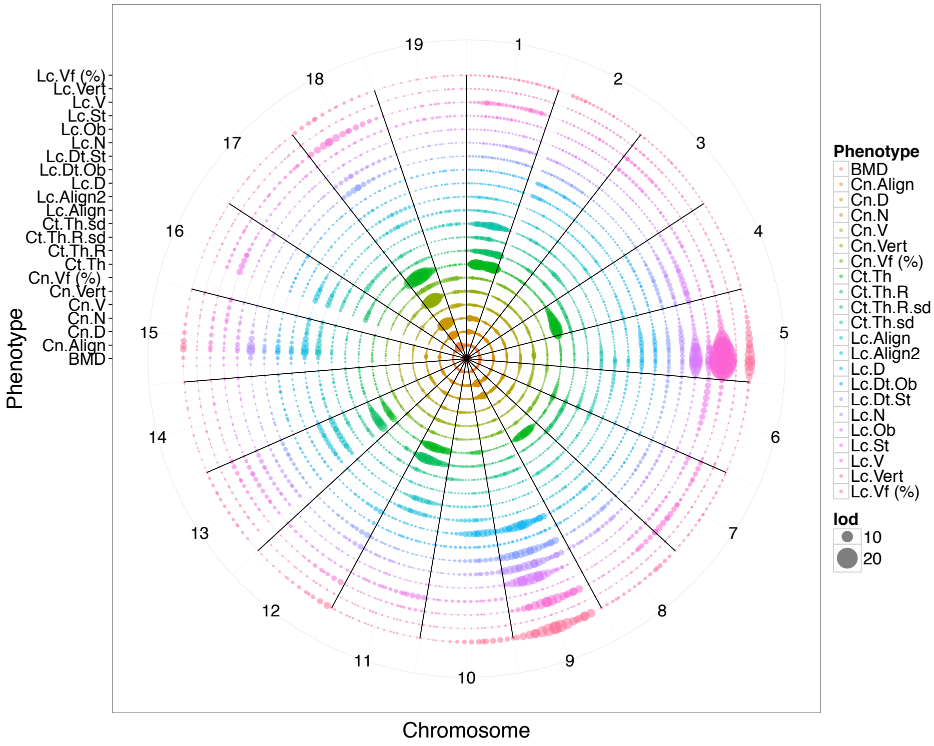

Figure S1 The graph shows the results from the single locus LOD scan performed on all of the phenotypes measured. The chromosomes are shown along the radial (θ) axis. Position within a chromosome is scaled by the maximum chromosome length and then used to plot the radial position more exactly. The radius and color are used to separate the different phenotypes. The size of the dot plotted indicates the LOD score for a single locus at this point.

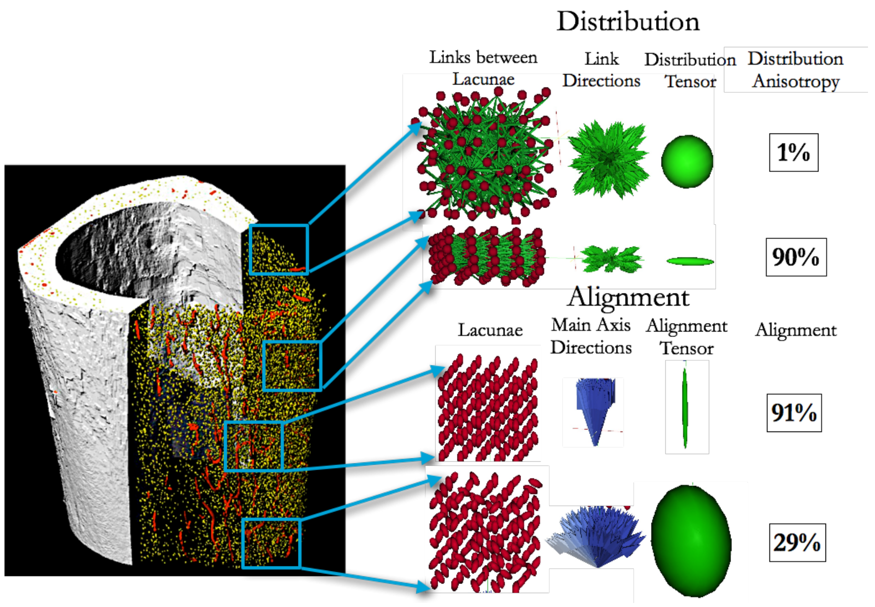

Figure S2 The figure illustrates the more complicated phenotypes analyzed, specifically alignment and distribution[24]. The distribution tensor (left upper panel) describes the spatial distribution of the lacunae, and whether or not they spacing is more sheet-like or stand-like. The alignment tensor (left lower panel) describes how the cells are oriented compared to each other. A high degree of anisotropy for this metric corresponds to very highly oriented systems.

Existing QTLs

The previously mapped QTLs were found using the QTL Archive run by the Churchill Group at the Jackson Laboratory (http://www.qtlarchive.org/). The entire database was filtered by taking only traits containing the words ”bone” or ”growth” which overlapped with the ranges calculated for the QTLs calculated in this study. The names, MGI Accession IDs and positions of the QTL are detailed in table S4 and plotted in figure S6. The MGI Accession IDs can be used to look up the reference to the specific QTL and the paper in which it was originally published using the Jackson.

Table S4: A list of all the previously identified bone and growth murine QTL which overlap with the ones found in this study. The first five columns show the chromsome, peak position, and starting and ending positions of the 95% confidence interval for each QTL as determined in this study. The fifth column shows the overlapping QTL from the QTLArchive. The shaded rows indicate QTL which do not overlap at all with BMD and are of particular interest for further investigation.

Chr.	Pos.(cM)	Start	End	Phen.	Overlapping QTL (MGI Accession ID)

Table S4: A list of all the previously identified bone and growth murine QTL which overlap with the ones found in this study. The first five columns show the chromsome, peak position, and starting and ending positions of the 95% confidence interval for each QTL as determined in this study. The fifth column shows the overlapping QTL from the QTLArchive. The shaded rows indicate QTL which do not overlap at all with BMD and are of particular interest for further investigation.

Chr.	Pos.(cM)	Start	End	Phen.	Overlapping QTL (MGI Accession ID)
1	60	19	73	Ct.Th.R.sd	bone mineral density (MGI:1349434), bone mineral density (MGI:3619533), bone mineral density (MGI:2389129), long bones (MGI:3639902), vertebral trabecular bone trait (MGI:3045043), vertebral trabecular bone trait (MGI:3045044), body growth early QTL (MGI:108576), body growth late QTL (MGI:108575), growth traits (MGI:3629098)
1	81	77	83	Lc.V	bone mineral density (MGI:3619534)
1	91	82	97	BMD	bone mineral density (MGI:3583577), total body bone mineral density (MGI:2668914)
1	93	83	101	Ct.Th.R	bone mineral density (MGI:3583577), total body bone mineral density (MGI:2668914)
3	44	26	62	Ct.Th.R.sd	bone density traits (MGI:2151120), bone mineral density (MGI:3694911), bone marrow pre-B (MGI:1332642), long bones (MGI:3639907), tibia bone quality traits (MGI:3721635)
4	59	47	71	BMD	femoral bone trait QTL (MGI:3701621), femoral bone morphometry (MGI:2154763), body growth early QTL (MGI:108558)
6	8	0	63	Lc.Dn	bone mineral density (MGI:3054099), bone mineral density (MGI:2389093), long bones (MGI:3639952)
6	14	4	30	Lc.St	bone mineral density (MGI:2389093)
7	2	2	12	BMD	long bones (MGI:3639954), modifier of mammary tumor growth (MGI:1890552)
7	2	2	68	Lc.St	bone density traits (MGI:2151122), bone length and organs (MGI:3639955), bone mineral density (MGI:2149339), bone mineral density (MGI:2178246), femoral bone morphometry (MGI:2154761), long bones (MGI:3639954), vertebral trabecular bone trait (MGI:3045046), body growth early QTL (MGI:108542), modifier of mammary tumor growth (MGI:1890552)

Table S4: A list of all the previously identified bone and growth murine QTL which overlap with the ones found in this study. The first five columns show the chromsome, peak position, and starting and ending positions of the 95% confidence interval for each QTL as determined in this study. The fifth column shows the overlapping QTL from the QTLArchive. The shaded rows indicate QTL which do not overlap at all with BMD and are of particular interest for further investigation.

Chr.	Pos.(cM)	Start	End	Phen.	Overlapping QTL (MGI Accession ID)
7	66	60	72	Ct.Th.R	femoral bone morphometry (MGI:2154761), vertebral trabecular bone trait (MGI:3045046)
7	66	40	72	Lc.V	bone length and organs (MGI:3639955), bone mineral density (MGI:2149339), femoral bone morphometry (MGI:2154761), vertebral trabecular bone trait (MGI:3045046), body growth early QTL (MGI:108542)
8	10	6	20	Lc.V	bone mineral density (MGI:3719112)
9	13	9	21	Ca.Dn	vertebral trabecular bone trait (MGI:3045048)
9	23	9	31	BMD	vertebral trabecular bone trait (MGI:3045048), vertebral trabecular bone trait (MGI:3045049)
9	35	25	39	Lc.V	vertebral trabecular bone trait (MGI:3045049), neonatal growth QTL (MGI:3640530)
9	41	31	51	Lc.Dn	bone mineral density (MGI:2389096), vertebral trabecular bone trait (MGI:3045050), neonatal growth QTL (MGI:3640530)
10	10	4	32	Lc.Dn	bone mechanical trait (MGI:3511309), femoral bone trait QTL (MGI:3701623)
11	6	2	20	Lc.St	bone mechanical trait (MGI:3511310), vertebral trabecular bone trait (MGI:3045053), body growth late QTL (MGI:108503)
11	46	40	52	Ct.Th.R	total body bone mineral density (MGI:2668918), body growth early QTL (MGI:108504), femur length in high growth mice (MGI:3698549)
11	55	32	70	Ct.Th.R.sd	bone mineral density (MGI:2389097), bone mineral density (MGI:3583579), total body bone mineral density (MGI:2668918), body growth early QTL (MGI:108504), femur length in high growth mice (MGI:3698549), weight gain in high growth mice (MGI:3698535)
12	15	5	21	Ct.Th.R	tibia bone quality traits (MGI:3721634)

Table S4: A list of all the previously identified bone and growth murine QTL which overlap with the ones found in this study. The first five columns show the chromsome, peak position, and starting and ending positions of the 95% confidence interval for each QTL as determined in this study. The fifth column shows the overlapping QTL from the QTLArchive. The shaded rows indicate QTL which do not overlap at all with BMD and are of particular interest for further investigation.

Chr.	Pos.(cM)	Start	End	Phen.	Overlapping QTL (MGI Accession ID)
13	46	12	58	Lc.St	bone mechanical trait (MGI:3511311), bone mineral density (MGI:2389099), bone length and organs (MGI:3639971), femoral bone trait QTL (MGI:3701625), tibia bone quality traits (MGI:3721636), vertebral trabecular bone trait (MGI:3045057), body growth late QTL (MGI:108493)
14	29	21	39	BMD	body growth early QTL (MGI:108480), body growth late QTL (MGI:1932377)
15	27	19	33	Lc.V	bone mineral density (MGI:1349437), vertebral trabecular bone trait (MGI:3045059)
17	44	34	52	Lc.V	bone mechanical trait (MGI:3511312), bone mineral density (MGI:3694913)
18	18	8	26	Lc.V	bone mineral density (MGI:2389103)
18	26	4	42	Ca.Dn	bone mineral density (MGI:2389103), vertebral trabecular bone trait (MGI:3045061)
18	40	18	48	BMD	bone mineral density (MGI:2389103), vertebral trabecular bone trait (MGI:3045061)

All Overlapping Markers

The following table S5 provide an overview of all the previously identified QTL in mice which overlap with the ranges determined for the given phenotypes. The table provides insight into possible mechanisms for these different QTL and might indicate multiple phenotypes being controlled by a single genetic locus. Specifically for the cellular phenotypes, which are relatively new in the bone field, the table provides insights into to which macro-scale these traits might be related. Specifically lacuna stretch (Lc.St) overlaps with "free running period" at Chr5 position 57cM potentially indicating a connection between running and lacuna shape. Furthermore the cellular phenotypes can be compared with other cellular or endocrine phenotypes to identify underlying mechanisms at this scale. For example lacuna density (Lc.Dn) and its overlap with obesity at Chr6 position 8cM. Finally these overlaps might further provide insight into the development cycle when specific traits like canal density (Ca.Dn) overlap with 10 week body weight, but not other weight metrics

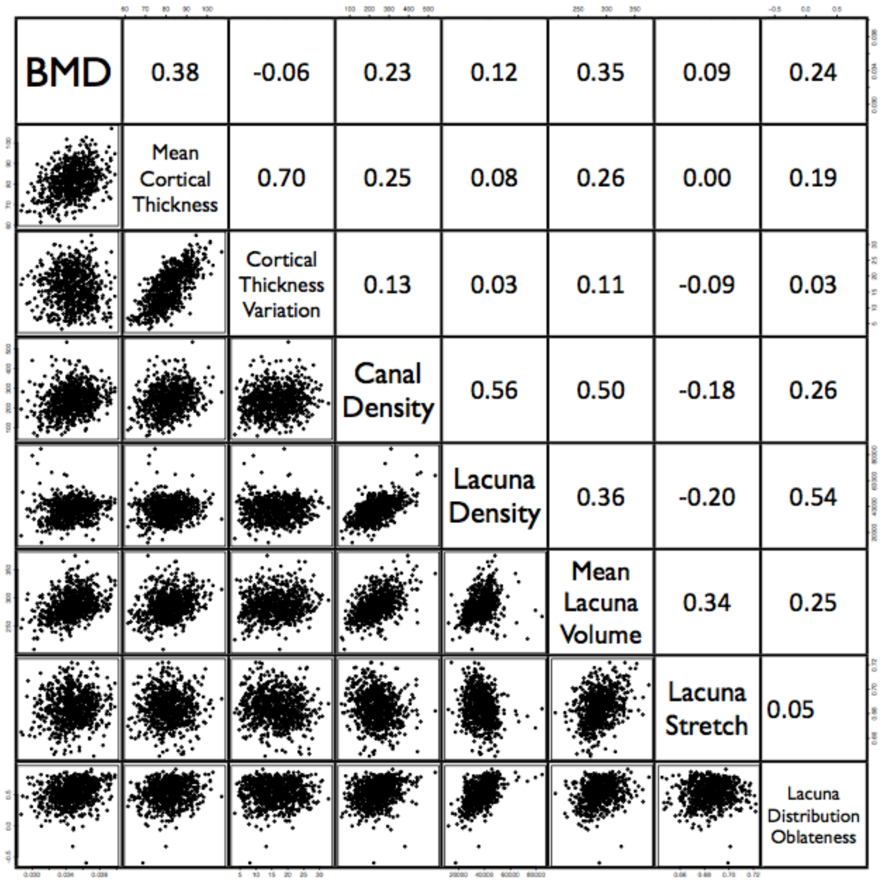

Figure S3 The graphs on the bottom half are shown for the selected metrics to show the relationship between them with the x-axis corresponding to the row and the y-axis to the column. This is quantified using the correlation coefficient shown for each metric pair in the upper half. Since highly correlated phenotypes are unlikely to show different genetic loci it is important that there are differences between the phenotypes chosen.

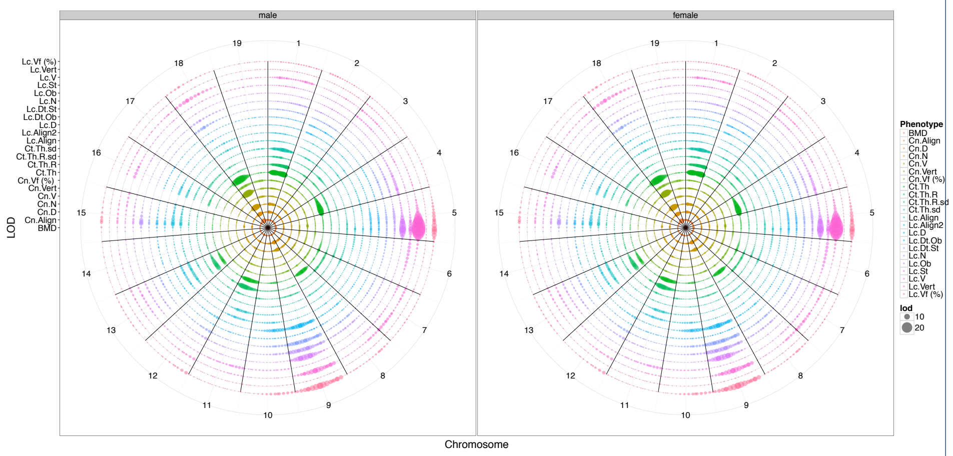

Figure S4 The graph shows the results from the single locus LOD scan performed on all of the phenotypes measured. The chromosomes are shown along the radial (θ) axis. Position within a chromosome is scaled by the maximum chromosome length and then used to plot the radial position more exactly. The radius and color are used to separate the different phenotypes. The size of the dot plotted indicates the LOD score for a single locus at this point. The entire dataset has been divided up into male (left) and female (right) and each analysis calculated separately in the corresponding graph.

indicating that this is a phenotype which is strongly determined at an early stage of development.

Table S5: A comprehensive list of all the previously identified murine QTL which overlap with the ones found in this study. The first five columns show the chromsome, peak position, and starting and ending positions of the 95% confidence interval for each QTL as determined in this study. The fifth column shows the overlapping QTL from the QTLArchive. The shaded rows indicate QTL which do not overlap at all with BMD and are of particular interest for further investigation.

Chr.	Pos.(cM)	Start	End	Phen.	Overlapping QTL (MGI Accession ID)
[redacted]	19	13	31	Ct.Th.R	darker modification of yellow agouti QTL (MGI:3051263), vertical (MGI:1858756)

Table S5: A comprehensive list of all the previously identified murine QTL which overlap with the ones found in this study. The first five columns show the chromsome, peak position, and starting and ending positions of the 95% confidence interval for each QTL as determined in this study. The fifth column shows the overlapping QTL from the QTLArchive. The shaded rows indicate QTL which do not overlap at all with BMD and are of particular interest for further investigation.

Chr.	Pos.(cM)	Start	End	Phen.	Overlapping QTL (MGI Accession ID)
1	60	19	73	Ct.Th.R.sd	activity-distance traveled (MGI:2150030), alcohol response (MGI:3053618), alcohol withdrawal (MGI:3037038), autoimmune ovarian dysgenesis (MGI:2670442), alcohol preference 1 (MGI:2152428), body growth early QTL (MGI:108576), body growth late QTL (MGI:108575), bone mineral density (MGI:1349434), bone mineral density (MGI:3619533), bone mineral density (MGI:2389129), body weight, QTL (MGI:3605813), body weight at necropsy (MGI:3639901), cocaine induced activation (MGI:3040196), cerebellum pattern fissures, declival (MGI:2151919), cholesterol QTL (MGI:2137601), dependence on morphine QTL (MGI:3510107), darker modification of yellow agouti QTL (MGI:3051263), darker modification of yellow agouti QTL (MGI:3051264), exploratory and excitability QTL (MGI:1314874), femur geometry (MGI:3512789), granulosa cell tumorigenesis (MGI:3574497), G protein deficiency-induced colitis (MGI:3573761), growth traits (MGI:3629098), hepatocarcinogenesis in females (MGI:3046664), horizontal distance (MGI:2680101), HDL QTL (MGI:2448347), HDL QTL (MGI:2448338), hematocrit QTL (MGI:3652905), heart rate quantitative locus (MGI:2662844), IgA nephropathy (MGI:3581246), insulin QTL (MGI:2148493), immunity to S. typhimurium (MGI:1891848), juvenile cystic kidney modifier (MGI:102752), long bones (MGI:3639902), limb length QTL (MGI:3042419), leishmaniasis resistance (MGI:2656541), loss of righting induced by ethanol (MGI:1096557), loss of righting induced by ethanol (MGI:2150637), light induced retinal degeneration (MGI:3042607), learning-contextual (MGI:2149002), lupus BXSB x NZW (MGI:3036323), lupus BXSB x NZW (MGI:3036325), mandible length (MGI:3528306), Mycobacterium bovis-induced systemic lupus erythematosus (MGI:2669461), melanoma modifier (MGI:1890553), Nidd5 on (MGI:3038860), non-insulin-dependent diabetes mellitus (MGI:1891281), novelty/stress induced locomotor activation (MGI:3580541), obesity QTL (MGI:2656143), obesity QTL (MGI:2150696), obesity and body weight QTL (MGI:3531516), organ weight QTL (MGI:3042410), pulmonary adenoma susceptibility (MGI:1345291), serum IGFBP-5 level QTL (MGI:3047361), skeletal size (tail length) (MGI:2149050), skin tumor susceptibility (MGI:1351472), skull morphology (MGI:2149680), skull morphology (MGI:2149681), systematic lupus erythematosus susceptibility (MGI:2149093), susceptibility to sialadenitis (MGI:2385531), susceptibility to tuberculosis (MGI:1888685), susceptibility to Salmonella typhimurium antigens (MGI:2661144), serum transfer induced arthritis (MGI:2151739), synechia (MGI:3052179), seizure susceptibility (MGI:1345649), tuberculosis resistance (MGI:2150671), total number errors, hindlimb coordination test (MGI:2151921), vertical (MGI:1858756), ventral midbrain iron content (MGI:3036599), vertebral morphology and mechanical traits (MGI:3697189), viral myocarditis susceptibility locus (MGI:3710942), vertebral trabecular bone trait (MGI:3045043), vertebral trabecular bone trait (MGI:3045044), white blood cell quantitative locus (MGI:3574405), white blood cell quantitative locus (MGI:3574408), white blood cell quantitative locus (MGI:3574409), weight (MGI:3608826)
1	81	77	83	Lc.V	activity response to ethanol (MGI:2151936), alcohol dependency (MGI:3050452), alcohol withdrawal (MGI:1309452), atherosclerosis (MGI:1890350), bone mineral density (MGI:3619534), caffeine metabolism QTL (MGI:2150827), cholesterol QTL (MGI:2137602), cholesterol QTL (MGI:3522038), fasting glucose (MGI:2660561), graft-versus-host disease (MGI:1346066), haloperidol induced catalepsy (MGI:2137474), melanoma modifier (MGI:1890554), modifier of (MGI:2684308), rotarod performance (MGI:2680094), systematic lupus erythematosus susceptibility (MGI:2149094), spermatocyte heat stress resistance (MGI:3579342), susceptibility to Salmonella typhimurium antigens (MGI:2661145), (MGI:3522039)

Table S5: A comprehensive list of all the previously identified murine QTL which overlap with the ones found in this study. The first five columns show the chromsome, peak position, and starting and ending positions of the 95% confidence interval for each QTL as determined in this study. The fifth column shows the overlapping QTL from the QTLArchive. The shaded rows indicate QTL which do not overlap at all with BMD and are of particular interest for further investigation.

Chr.	Pos.(cM)	Start	End	Phen.	Overlapping QTL (MGI Accession ID)
1	91	82	97	BMD	bone mineral density (MGI:3583577), body weight at 8 weeks QTL (MGI:2681283), femur breaking strength (MGI:1891230), HDL QTL (MGI:2679572), HDL QTL (MGI:2448339), modifier of obesity related sterility (MGI:2149822), systematic lupus erythematosus susceptibility (MGI:1931844), systematic lupus erythematosus susceptibility (MGI:2149094), spermatocyte heat stress resistance (MGI:3579342), total body bone mineral density (MGI:2668914), zinc induced tolerance (MGI:3588721)
1	93	83	101	Ct.Th.R	bone mineral density (MGI:3583577), body weight at 8 weeks QTL (MGI:2681283), femur breaking strength (MGI:1891230), HDL QTL (MGI:2679572), HDL QTL (MGI:2448339), modifier of obesity related sterility (MGI:2149822), autoimmune orchitis resistance (MGI:108003), systematic lupus erythematosus susceptibility (MGI:1931844), total body bone mineral density (MGI:2668914), zinc induced tolerance (MGI:3588721)
3	44	26	62	Ct.Th.R.sd	alcohol preference 6 (MGI:2152433), arthropathy in MRL and DBA/1 mice (MGI:3588384), atherosclerotic lesion area (MGI:3613543), bone density traits (MGI:2151120), bone mineral density (MGI:3694911), bone marrow pre-B (MGI:1332642), body weight QTL (MGI:3715735), cholesterol QTL (MGI:2137607), C. trachomatis resistance QTL (MGI:3692642), G protein deficiency-induced colitis (MGI:3573760), hematocrit QTL (MGI:3622212), HDL level (MGI:3036902), HDL QTL (MGI:3041283), induction of brown adipocytes (MGI:2149993), insulin dependent diabetes susceptibility (MGI:96405), long bones (MGI:3639907), liver iron content (MGI:3036610), mandible length (MGI:3528308), modifier of obesity related sterility (MGI:2149823), Nakano cataract modifier (MGI:2451367), novelty/stress induced locomotor activation (MGI:3580542), novelty/stress induced locomotor activation (MGI:3580543), organ weight (MGI:3639908), platelet activation (MGI:1100875), skull morphology (MGI:2149683), systematic lupus erythematosus susceptibility (MGI:2149097), systemic lupus erythmatosus suppressor (MGI:1353463), susceptibility to Salmonella typhimurium antigens (MGI:3510707), suppressor of superoxide (MGI:2152196), tibia bone quality traits (MGI:3721635), tuberculosis severity (MGI:1345631), total cholesterol level (MGI:3038481), T cell ratio modifier QTL (MGI:3042195), T cell ratio modifier QTL (MGI:3042196), tooth shape (MGI:2386694), vertebral morphology and mechanical traits (MGI:3697206), white blood cell quantitative locus (MGI:3574406)
4	59	47	71	BMD	aromatase activity QTL (MGI:1196361), aromatase activity QTL (MGI:1196363), alloantigen response (MGI:2150757), autoimmune renal vasculitis (MGI:2149547), atherosclerosis susceptibility QTL (MGI:1933987), body growth early QTL (MGI:108558), beta-carboline-induced seizures (MGI:104794), body weight at 8 weeks QTL (MGI:2681284), caffeine metabolism QTL (MGI:2150828), femoral bone trait QTL (MGI:3701621), fecundity QTL (MGI:1329007), femoral bone morphometry (MGI:2154763), gastritis type A susceptibility locus (MGI:1346075), hepatocarcinogen resistance (MGI:3046736), insulin dependent diabetes susceptibility (MGI:3603297), insulin dependent diabetes susceptibility (MGI:96411), immunoregulatory (MGI:2149679), lupus NZB x NZW (MGI:105965), lithogenic gene (MGI:2653453), lupus in MRL and B6 F2 cross, QTL (MGI:1203738), lung tumor shape-determining (MGI:2664841), skin tumor susceptibility (MGI:1351479), skull morphology (MGI:2149686), systemic lupus erythmatosus suppressor (MGI:1353462), susceptibility to lung cancer (MGI:2387365), susceptibility to lung cancer (MGI:1335085), small testis weight (MGI:3654113), startle response (MGI:1858759), soft tissue heal (MGI:2176908), SGC/Knj cross B6 QTL (MGI:3624658), tooth shape (MGI:2386696), vertebral morphology and mechanical traits (MGI:3697192), weight (MGI:3608828)

Table S5: A comprehensive list of all the previously identified murine QTL which overlap with the ones found in this study. The first five columns show the chromsome, peak position, and starting and ending positions of the 95% confidence interval for each QTL as determined in this study. The fifth column shows the overlapping QTL from the QTLArchive. The shaded rows indicate QTL which do not overlap at all with BMD and are of particular interest for further investigation.

Chr.	Pos.(cM)	Start	End	Phen.	Overlapping QTL (MGI Accession ID)
5	53	45	53	Lc.V	age of eyelid (MGI:1858757), body weight day 30 males (MGI:1858761), cocaine related behavior (MGI:2150992), cerebellum pattern fissures, declival (MGI:2151922), cytokine production (MGI:1333845), dopamine receptor binding (MGI:2150994), dorsal hippocampal volume (MGI:3696435), high density lipoprotein (HDL) level (MGI:2149552), HDL level (MGI:3514360), HDL QTL (MGI:2387135), heterogeneity of muscle Gapd decay (MGI:3521868), hypercapnic duty (MGI:2682394), lymph node cytotoxic T lymphocyte percentage (MGI:3582971), lipoprotein QTL (MGI:3511265), skeletal muscle weight (MGI:3640571), vertebral morphology and mechanical traits (MGI:3697194)
5	67	57	73	Lc.St	autoimmune extremity vasculitis in MRL mice (MGI:2680907), body weight females and males day (MGI:1858760), correlation in cytokine production (MGI:1333852), dextran sodium sulfate induced colitis (MGI:1345666), fat induced diabetes (MGI:1891121), femur geometry (MGI:3512916), free running period (MGI:2151078), (MGI:2664783), lithogenic gene (MGI:3576867), methamphetamine response QTL (MGI:3590510), nurturing ability QTL (MGI:2179258), small effect CIA locus (MGI:3717842), weight (MGI:3608829)
5	68	65	69	Lc.V	free running period (MGI:2151078), (MGI:2664783), nurturing ability QTL (MGI:2179258)
6	8	0	63	Lc.Dn	acute lung injury QTL (MGI:2178585), atherosclerosis (MGI:3618817), bronchial hyperresponsiveness (MGI:1351272), bitterness sensitivity (MGI:2150764), bone mineral density (MGI:3054099), bone mineral density (MGI:2389093), bladder tumor susceptibility (MGI:1934674), bitterness (MGI:3606253), body weight QTL (MGI:1932503), cocaine induced activation (MGI:3040199), circadian period of locomotor activity (MGI:3588344), femoral cross-sectional area (MGI:2668935), fasting (MGI:2149370), femur geometry (MGI:3512918), gastritis type A susceptibility locus (MGI:2176188), granule cell layer (MGI:3052078), HDL QTL (MGI:2448344), HDL QTL (MGI:3041285), immediate cutaneous hypersensitivity (MGI:1861748), IGF-1 serum levels (MGI:3046873), immunoregulatory (MGI:3032855), impaired glucose tolerance (MGI:3038478), long bones (MGI:3639952), lithogenic gene (MGI:2653455), leishmaniasis resistance (MGI:2656510), lung tumor shape-determining (MGI:2664845), lupus BXSB x NZW (MGI:3036208), multigenic obesity QTL (MGI:99506), modifier of ocular retardation (MGI:3656497), modifier of Salmonella typhimurium susceptibility (MGI:3690347), non-HDL QTL (MGI:2686904), novelty/stress induced locomotor activation (MGI:3580545), obesity QTL (MGI:2150703), obesity and body weight QTL (MGI:3531518), organ weight (MGI:3639951), ovulation rate QTL (MGI:1196401), ovarian teratoma susceptibility (MGI:109357), plasma apolipoprotein B (human) regulator (MGI:2137387), pulmonary adenoma susceptibility (MGI:1345294), skeletal size (tail length) (MGI:2149052), skin tumor susceptibility (MGI:1351483), skull morphology (MGI:2149688), susceptibility to lung cancer (MGI:2155622), susceptibility to lung cancer (MGI:1335097), segregation of mitochondrial DNA QTL (MGI:2179704), susceptibility to Salmonella typhimurium antigens (MGI:2661146), soft tissue heal (MGI:2176909), SGC/Knj cross B6 QTL (MGI:3624752), T cell receptor beta variable 4, control (MGI:1927661)
6	14	4	30	Lc.St	acute lung injury QTL (MGI:2178585), bone mineral density (MGI:2389093), bladder tumor susceptibility (MGI:1934674), cocaine induced activation (MGI:3040199), circadian period of locomotor activity (MGI:3588344), fasting (MGI:2149370), granule cell layer (MGI:3052078), HDL QTL (MGI:3041285), impaired glucose tolerance (MGI:3038478), leishmaniasis resistance (MGI:2656510), lupus BXSB x NZW (MGI:3036208), multigenic obesity QTL (MGI:99506), novelty/stress induced locomotor activation (MGI:3580545), organ weight (MGI:3639951), ovulation rate QTL (MGI:1196401), skull morphology (MGI:2149688), susceptibility to lung cancer (MGI:1335097), segregation of mitochondrial DNA QTL (MGI:2179704), SGC/Knj cross B6 QTL (MGI:3624752)
6	21	10	21	BMD	bladder tumor susceptibility (MGI:1934674), cocaine induced activation (MGI:3040199), fasting (MGI:2149370), susceptibility to lung cancer (MGI:1335097)

Table S5: A comprehensive list of all the previously identified murine QTL which overlap with the ones found in this study. The first five columns show the chromsome, peak position, and starting and ending positions of the 95% confidence interval for each QTL as determined in this study. The fifth column shows the overlapping QTL from the QTLArchive. The shaded rows indicate QTL which do not overlap at all with BMD and are of particular interest for further investigation.

Chr.	Pos.(cM)	Start	End	Phen.	Overlapping QTL (MGI Accession ID)
6	58	46	63	BMD	atherosclerosis (MGI:3618817), bronchial hyperresponsiveness (MGI:1351272), bitterness (MGI:3606253), femur geometry (MGI:3512918), gastritis type A susceptibility locus (MGI:2176188), HDL QTL (MGI:2448344), immediate cutaneous hypersensitivity (MGI:1861748), IGF-1 serum levels (MGI:3046873), lung tumor shape-determining (MGI:2664845), modifier of ocular retardation (MGI:3656497), obesity and body weight QTL (MGI:3531518), plasma apolipoprotein B (human) regulator (MGI:2137387), susceptibility to lung cancer (MGI:2155622)
6	63	54	63	Ct.Th.R	bitterness (MGI:3606253), femur geometry (MGI:3512918), lung tumor shape-determining (MGI:2664845), modifier of ocular retardation (MGI:3656497), susceptibility to lung cancer (MGI:2155622)
6	63	61	63	Ct.Th.R.sd	lung tumor shape-determining (MGI:2664845)
7	2	2	12	BMD	allergen-induced bronchial hyperresponsiveness (MGI:2148588), Avp transcript abundance QTL (MGI:3580594), Crhr1 transcript abundance QTL (MGI:3580597), long bones (MGI:3639954), lupus BXSB x NZW (MGI:3036326), mandible length (MGI:3528312), mandible size (MGI:2148836), modifier of mammary tumor growth (MGI:1890552), maternal (MGI:2661774), susceptibility to lung cancer (MGI:2387375), ventricular size (MGI:2664092)
7	2	2	68	Lc.St	allergen-induced bronchial hyperresponsiveness (MGI:2148588), adiposity (MGI:2149043), anti-erythrocyte autoantibody modifier (MGI:2137852), angle of entrainment (MGI:2151079), aortic lesion size (MGI:3036905), alcohol preference 7 (MGI:2155164), Avp transcript abundance QTL (MGI:3580594), bone density traits (MGI:2151122), body growth early QTL (MGI:108542), bronchial hyperresponsiveness (MGI:2150651), beta-carboline induced seizures (MGI:1343055), bone length and organs (MGI:3639955), bone mineral density (MGI:2149339), bone mineral density (MGI:2178246), cocaine induced activation (MGI:3040200), consumption-saccharin intake (MGI:2148652), Crhr1 transcript abundance QTL (MGI:3580597), dsRNA-induced UCM (MGI:3055206), ether anesthesia (MGI:3522104), ethanol induced thermoregulation (MGI:3044650), femoral cross-sectional area (MGI:2668940), femoral bone morphometry (MGI:2154761), femur geometry (MGI:3512919), granular brain (MGI:3047352), hepatocarcinogenesis susceptibility (MGI:96056), HDL level (MGI:3514361), HDL cholesterol level (MGI:3528033), wound healing/regeneration (MGI:2148578), hematocrit QTL (MGI:3652907), Hfe modifier (MGI:3692625), insulin dependent diabetes susceptibility (MGI:3608893), long bones (MGI:3639954), limb length QTL (MGI:3042421), lipoprotein QTL (MGI:3511880), lung tumor shape-determining (MGI:2664842), lupus BXSB x NZW (MGI:3036326), mandible shape (MGI:2148817), mandible length (MGI:3528312), mandible length (MGI:3528313), mandible size (MGI:2148836), mean corpuscular volume QTL (MGI:3622214), modifier of (MGI:3576647), modifier of muscularity (MGI:3054067), modifier of mammary tumor growth (MGI:1890552), modifier of mammary tumor progression (MGI:3043750), multigenic obesity QTL (MGI:99505), maternal (MGI:2661774), New Zealand Black autoimmunity (MGI:3530666), non-HDL QTL (MGI:2686905), nicotine induced locomotor activity (MGI:3629971), obesity QTL (MGI:2150704), organ weight (MGI:3639956), osteosarcoma susceptibility (MGI:3577289), radiation induced gastroschisis (MGI:1330298), salmonella enteritidis susceptibility (MGI:2664974), skin tumor susceptibility (MGI:104788), skull morphology (MGI:2149692), skull morphology (MGI:2149690), susceptibility to lung cancer (MGI:2387375), Streptococcus pneumoniae infection resistance (MGI:2661190), spermatogenesis defect (MGI:3654117), susceptibility to sialadenitis (MGI:2385533), soft tissue heal (MGI:2176910), seizure severity (MGI:1098565), TallyHo associated body (MGI:1932202), tuberculosis resistance (MGI:2150673), T cell receptor induced activation (MGI:1343127), T cell secretion of IL4 QTL (MGI:3590286), ventricular size (MGI:2664092), ventral midbrain iron content (MGI:3036602), VPA induced neural tube (MGI:3043719), vertebral trabecular bone trait (MGI:3045046)

Table S5: A comprehensive list of all the previously identified murine QTL which overlap with the ones found in this study. The first five columns show the chromsome, peak position, and starting and ending positions of the 95% confidence interval for each QTL as determined in this study. The fifth column shows the overlapping QTL from the QTLArchive. The shaded rows indicate QTL which do not overlap at all with BMD and are of particular interest for further investigation.

Chr.	Pos.(cM)	Start	End	Phen.	Overlapping QTL (MGI Accession ID)
7	66	60	72	Ct.Th.R	alcohol preference locus 12, male (MGI:1858391), alcohol preference locus 14, female (MGI:1858393), determination of interleukin 4 commitment (MGI:2149368), ethanol induced thermoregulation (MGI:3044650), femoral bone morphometry (MGI:2154761), HDL cholesterol level (MGI:3528033), hematocrit QTL (MGI:3652907), IL-4 producing potential (MGI:3702119), multigenic obesity QTL (MGI:99505), nicotine induced locomotor activity (MGI:3629971), susceptibility to lung cancer (MGI:2387363), seizure severity (MGI:1098565), T cell secretion of IL4 QTL (MGI:3590286), VPA induced neural tube (MGI:3043719), vertebral trabecular bone trait (MGI:3045046)
7	66	40	72	Lc.V	adiposity (MGI:2149043), anti-erythrocyte autoantibody modifier (MGI:2137852), alcohol preference locus 12, male (MGI:1858391), alcohol preference locus 14, female (MGI:1858393), angle of entrainment (MGI:2151079), body growth early QTL (MGI:108542), bone length and organs (MGI:3639955), bone mineral density (MGI:2149339), consumption-saccharin intake (MGI:2148652), determination of interleukin 4 commitment (MGI:2149368), ether anesthesia (MGI:3522104), ethanol induced thermoregulation (MGI:3044650), femoral bone morphometry (MGI:2154761), hepatocarcinogenesis susceptibility (MGI:96056), HDL cholesterol level (MGI:3528033), wound healing/regeneration (MGI:2148578), hematocrit QTL (MGI:3652907), insulin dependent diabetes susceptibility (MGI:3608893), IL-4 producing potential (MGI:3702119), limb length QTL (MGI:3042421), lung tumor shape-determining (MGI:2664842), mandible shape (MGI:2148817), mandible length (MGI:3528313), multigenic obesity QTL (MGI:99505), New Zealand Black autoimmunity (MGI:3530666), nicotine induced locomotor activity (MGI:3629971), obesity QTL (MGI:2150704), organ weight (MGI:3639956), salmonella enteritidis susceptibility (MGI:2664974), skull morphology (MGI:2149692), skull morphology (MGI:2149690), susceptibility to lung cancer (MGI:2387363), susceptibility to sialadenitis (MGI:2385533), soft tissue heal (MGI:2176910), seizure severity (MGI:1098565), T cell receptor induced activation (MGI:1343127), T cell secretion of IL4 QTL (MGI:3590286), VPA induced neural tube (MGI:3043719), vertebral trabecular bone trait (MGI:3045046)
8	10	6	20	Lc.V	alcohol withdrawal (MGI:3037039), alcohol preference 8 (MGI:2155165), body (MGI:3528034), bone mineral density (MGI:3719112), lung tumor shape-determining (MGI:2664840), non-heme iron levels (MGI:3691600), nicotine induced locomotor activity (MGI:3629973), susceptibility to lung cancer (MGI:2387364)
9	13	9	21	Ca.Dn	alcohol preference QTL (MGI:3652895), castaneus 10 week body weight (MGI:2137239), cholesterol QTL (MGI:3624134), haloperidol-induced catelepsy (MGI:2148581), IgA nephropathy (MGI:3581247), body length (MGI:2656815), leishmaniasis resistance (MGI:3625867), morphine antinociception (MGI:2151859), organ weight QTL (MGI:3042416), serum IGFBP-5 level QTL (MGI:3047362), skull morphology (MGI:2149694), susceptibility to lung cancer (MGI:1335074), tuberculosis severity (MGI:1345641), vertebral trabecular bone trait (MGI:3045048)

Table S5: A comprehensive list of all the previously identified murine QTL which overlap with the ones found in this study. The first five columns show the chromsome, peak position, and starting and ending positions of the 95% confidence interval for each QTL as determined in this study. The fifth column shows the overlapping QTL from the QTLArchive. The shaded rows indicate QTL which do not overlap at all with BMD and are of particular interest for further investigation.

Chr.	Pos.(cM)	Start	End	Phen.	Overlapping QTL (MGI Accession ID)
9	23	9	31	BMD	activity-distance traveled (MGI:2150033), activity response to ethanol (MGI:3694112), alopecia areata (MGI:2677751), alcohol preference QTL (MGI:3652893), alcohol preference QTL (MGI:3652895), alcohol preference 5 (MGI:2152432), APP associated premature death (MGI:3663090), castaneus 10 week body weight (MGI:2137239), cocaine induced activation (MGI:3040201), cocaine related behavior (MGI:2150997), cholesterol QTL (MGI:3624134), cholesterol QTL (MGI:3624135), ethanol consumption (MGI:1858453), fecundity QTL (MGI:3640532), HDL QTL (MGI:2656139), haloperidol-induced catelepsy (MGI:2148581), hypothermia (MGI:2150693), IgA nephropathy (MGI:3581247), body length (MGI:2656815), leishmaniasis resistance (MGI:3625867), leishmaniasis resistance (MGI:1277141), morphine antinociception (MGI:2151859), obesity QTL (MGI:1349407), organ weight QTL (MGI:3042416), serum IGFBP-5 level QTL (MGI:3047362), serum IGFBP-5 level QTL (MGI:3047363), skull morphology (MGI:2149694), susceptibility to lung cancer (MGI:1335074), body temperature response to dietary restriction, QTL (MGI:2655235), tuberculosis severity (MGI:1345641), vertebral trabecular bone trait (MGI:3045048), vertebral trabecular bone trait (MGI:3045049)
9	35	25	39	Lc.V	activity-distance traveled (MGI:2150033), activity response to ethanol (MGI:3694112), alcohol preference QTL (MGI:3652893), alcohol preference 5 (MGI:2152432), atherosclerosis (MGI:3613552), body length (MGI:2446731), caffeine metabolism QTL (MGI:2150829), cocaine related behavior (MGI:2150997), cholesterol QTL (MGI:3624135), dermititis (MGI:1890944), ethanol consumption (MGI:1858453), fecundity QTL (MGI:3640532), hypothermia (MGI:2150693), inflammatory bowel disease QTL (MGI:3574489), body length (MGI:2656816), modifier of mammary tumor progression (MGI:3043752), neonatal growth QTL (MGI:3640530), serum IGFBP-5 level QTL (MGI:3047363), susceptibility to Salmonella typhimurium antigens (MGI:3510709), soft tissue heal (MGI:2176913), body temperature response to dietary restriction, QTL (MGI:2655235), triglyceride QTL (MGI:3029249), vertebral trabecular bone trait (MGI:3045049)
9	41	31	51	Lc.Dn	adiposity (MGI:2149045), atherosclerosis (MGI:3613552), body length (MGI:2446731), bleomycin-induced pulmonary fibrosis (MGI:3606258), bone mineral density (MGI:2389096), caffeine metabolism QTL (MGI:2150829), dermititis (MGI:1890944), ethanol conditioned taste aversion (MGI:2148662), femur breaking strength (MGI:2386571), femur geometry (MGI:3512925), G protein deficiency-induced colitis (MGI:3573762), haloperidol-induced catalepsy (MGI:2137472), inflammatory bowel disease QTL (MGI:3574489), keratinocyte stem cell locus (MGI:2668899), body length (MGI:2656816), life span (MGI:2179244), lithogenic gene (MGI:3577214), lithogenic gene (MGI:2176161), learning contextual (MGI:2148270), modifier of mammary tumor progression (MGI:3043752), multigenic obesity QTL (MGI:1890670), neonatal growth QTL (MGI:3640530), pulmonary adenoma susceptibility (MGI:108510), skin tumor susceptibility (MGI:1351478), skull morphology (MGI:2149695), susceptibility to Salmonella typhimurium antigens (MGI:3510709), soft tissue heal (MGI:2176913), streptozotocin induced diabetes susceptibility (MGI:3529674), taste bitterness sensitivity (MGI:2148969), tri-nitrobenzene sulfonic acid susceptible (MGI:2451075), triglyceride QTL (MGI:3029249), vertebral morphology and mechanical traits (MGI:3697204), vertebral trabecular bone trait (MGI:3045050)
10	10	4	32	Lc.Dn	amphetamine distance traveled (MGI:3695446), atherosclerotic lesion area (MGI:3613540), atherosclerotic lesion area (MGI:3613541), atherosclerosis (MGI:2451366), barrel field size (MGI:3654070), bone mechanical trait (MGI:3511309), femoral bone trait QTL (MGI:3701623), IgA nephropathy (MGI:3581245), lithogenic gene (MGI:3577215), myeloperoxidase-specific anti-neutrophil cytoplasmic autoantibody QTL (MGI:3624755), SGC/Knj cross B6 QTL (MGI:3624754), UVB induced immunosuppression (MGI:2150915)

Table S5: A comprehensive list of all the previously identified murine QTL which overlap with the ones found in this study. The first five columns show the chromsome, peak position, and starting and ending positions of the 95% confidence interval for each QTL as determined in this study. The fifth column shows the overlapping QTL from the QTLArchive. The shaded rows indicate QTL which do not overlap at all with BMD and are of particular interest for further investigation.

Chr.	Pos.(cM)	Start	End	Phen.	Overlapping QTL (MGI Accession ID)
11	6	2	20	Lc.St	body growth late QTL (MGI:108503), bitterness sensitivity (MGI:2150765), bone mechanical trait (MGI:3511310), femur geometry (MGI:3512926), glucose homeostasis QTL (MGI:3046234), hematocrit QTL (MGI:3652908), hippocampal weight (MGI:3052079), IGF-1 serum levels (MGI:3046876), lithogenic gene (MGI:3577226), leishmaniasis resistance (MGI:2656512), mandible length (MGI:3528315), mean corpuscular volume QTL (MGI:3622215), mandible length (MGI:2663054), non-insulin-dependent diabetes mellitus 4 in (MGI:1355302), nicotine induced locomotor activity (MGI:3629965), organ weight QTL (MGI:3042417), skull morphology (MGI:2149697), tooth shape (MGI:2386699), vertebral trabecular bone trait (MGI:3045053), weight 6 weeks QTL (MGI:3527566)
11	46	40	52	Ct.Th.R	alcohol preference locus 2, female (MGI:107452), body growth early QTL (MGI:108504), body weight QTL (MGI:1316681), body weight at necropsy (MGI:3639968), ethanol conditioned taste aversion (MGI:2148663), femur length in high growth mice (MGI:3698549), graft-versus host disease (MGI:2386605), HDL level (MGI:3514362), HDL QTL (MGI:3607077), insulin dependent diabetes susceptibility (MGI:96406), immunity to S. typhimurium (MGI:3762327), kidney weight QTL (MGI:3698539), body length (MGI:2656817), lethal factor toxin susceptibility (MGI:3609214), liver weight QTL (MGI:3698548), modifier of Trp53 (MGI:3575566), modifier of Salmonella typhimurium susceptibility (MGI:3690348), nicotine induced locomotor activity (MGI:3629966), pulmonary adenoma susceptibility (MGI:109195), serum IGFBP-5 level QTL (MGI:3047366), skeletal size (tail length) (MGI:2149054), systematic lupus erythematosus susceptibility (MGI:2149722), semilethal (MGI:2148365), spermatocyte heat stress resistance (MGI:3579343), susceptibility to Salmonella typhimurium antigens (MGI:2661147), total body bone mineral density (MGI:2668918), tooth shape (MGI:2386700), ventral midbrain iron content (MGI:3036604)
11	55	32	70	Ct.Th.R.sd	alcohol preference locus 2, female (MGI:107452), acute lung injury QTL (MGI:1345621), body growth early QTL (MGI:108504), bone mineral density (MGI:2389097), bone mineral density (MGI:3583579), body weight QTL (MGI:1316681), body weight at necropsy (MGI:3639968), castaneus 10 week body weight (MGI:2137240), compensatory renal hypertrophy QTL (MGI:1298385), C. trachomatis resistance QTL (MGI:3692643), ethanol induced thermoregulation (MGI:3044651), ethanol conditioned taste aversion (MGI:2148663), femur length in high growth mice (MGI:3698549), fat induced adiposity (MGI:3707782), graft-versus host disease (MGI:2386605), HDL level (MGI:3514362), HDL QTL (MGI:3607077), wound healing/regeneration (MGI:2669605), insulin dependent diabetes susceptibility (MGI:96406), immunity to S. typhimurium (MGI:3762327), kidney weight QTL (MGI:3698539), body length (MGI:2656817), leishmaniasis resistance (MGI:2656607), lethal factor toxin susceptibility (MGI:3609213), lethal factor toxin susceptibility (MGI:3609214), liver weight QTL (MGI:3698548), mandible length (MGI:3528316), modifier of Trp53 (MGI:3575566), modifier of Salmonella typhimurium susceptibility (MGI:3690348), non-insulin-dependent diabetes mellitus 1 in (MGI:1355320), nicotine induced locomotor activity (MGI:3629966), organ weight (MGI:3639967), pulmonary adenoma susceptibility (MGI:109195), pulmonary adenoma susceptibility (MGI:1345383), pulmonary adenoma susceptibility (MGI:1345295), serum IGFBP-5 level QTL (MGI:3047366), skeletal size (tail length) (MGI:2149054), skull morphology (MGI:2149698), systematic lupus erythematosus susceptibility (MGI:2149722), semilethal (MGI:2148365), spermatocyte heat stress resistance (MGI:3579343), susceptibility to Salmonella typhimurium antigens (MGI:2661147), streptozotocin induced diabetes (MGI:3528282), tail length QTL (MGI:3698541), total body bone mineral density (MGI:2668918), tooth shape (MGI:2386700), ventral midbrain iron content (MGI:3036604), vertebral morphology and mechanical traits (MGI:3697200), weight gain in high growth mice (MGI:3698535)

Table S5: A comprehensive list of all the previously identified murine QTL which overlap with the ones found in this study. The first five columns show the chromsome, peak position, and starting and ending positions of the 95% confidence interval for each QTL as determined in this study. The fifth column shows the overlapping QTL from the QTLArchive. The shaded rows indicate QTL which do not overlap at all with BMD and are of particular interest for further investigation.

Chr.	Pos.(cM)	Start	End	Phen.	Overlapping QTL (MGI Accession ID)
12	3	3	13	Lc.Dn	atherosclerotic lesion area (MGI:3613544), fluctuating asymmetry QTL (MGI:1098213), femoral cross-sectional area (MGI:2668941), limb length QTL (MGI:3042423), NZB and NZW autoimmunity (MGI:3033344)
12	3	3	11	Lc.V	atherosclerotic lesion area (MGI:3613544), fluctuating asymmetry QTL (MGI:1098213), femoral cross-sectional area (MGI:2668941), limb length QTL (MGI:3042423), NZB and NZW autoimmunity (MGI:3033344)
12	15	5	21	Ct.Th.R	atherosclerotic lesion area (MGI:3613544), atherosclerosis (MGI:2451364), Avp transcript abundance QTL (MGI:3580595), body weight at necropsy (MGI:3639969), circadian period of locomotor activity (MGI:3588345), etomidate (MGI:2663494), fluctuating asymmetry QTL (MGI:1098213), femoral cross-sectional area (MGI:2668941), fatty liver 1 in (MGI:3723314), HDL QTL (MGI:2656140), limb length QTL (MGI:3042423), NZB and NZW autoimmunity (MGI:3033344), skin tumor susceptibility (MGI:1351480), tibia bone quality traits (MGI:3721634)
13	12	10	18	Ct.Th.R	(MGI:2682418), modifier of Salmonella typhimurium susceptibility (MGI:3690349), systolic blood pressure (MGI:2684017), voluntary alcohol consumption QTL (MGI:3590250)
13	46	12	58	Lc.St	Angiostrongylus costaricensis nematode susceptibility (MGI:2389043), Avp transcript abundance QTL (MGI:3580596), body growth late QTL (MGI:108493), bone mechanical trait (MGI:3511311), bone mineral density (MGI:2389099), bone length and organs (MGI:3639971), body weight day 30 males (MGI:1858763), castaneus 10 week body weight (MGI:2137241), cocaine induced activation (MGI:3040202), cerebellum pattern fissures, declival (MGI:2151924), circadian period of locomotor activity (MGI:3588346), Crh transcript abundance QTL (MGI:3580593), delta power in slow-wave sleep (MGI:2135996), femoral bone trait QTL (MGI:3701625), HDL cholesterol level (MGI:3528035), wound healing/regeneration (MGI:2148579), wound healing/regeneration (MGI:2148580), hippocampal weight (MGI:3052076), (MGI:3528036), (MGI:2682418), insulin dependent diabetes susceptibility (MGI:99416), lipoprotein QTL (MGI:2662493), listeriosis resistance (MGI:2150909), lithogenic gene (MGI:3576865), mandible length (MGI:3528319), molar crown diameter QTL (MGI:3706141), modifier of obesity (MGI:3037101), modifier of Salmonella typhimurium susceptibility (MGI:3690349), pulmonary adenoma susceptibility (MGI:1345293), salmonella enteritidis susceptibility (MGI:3588678), skeletal size (tail length) (MGI:2149055), skull morphology (MGI:2149703), susceptibility to lung cancer (MGI:2387368), spermatogenesis defect (MGI:3654118), systolic blood pressure (MGI:2684017), tibia bone quality traits (MGI:3721636), voluntary alcohol consumption QTL (MGI:3590250), vertebral trabecular bone trait (MGI:3045057)
14	29	21	39	BMD	atherosclerosis (MGI:1890354), body growth early QTL (MGI:108480), body growth late QTL (MGI:1932377), body weight (MGI:3707783), heart weight quantitative locus (MGI:2662846), lean body mass (MGI:2656811), modifier of obesity related sterility (MGI:2149824), myocardial infarction (MGI:3625042), non-insulin-dependent diabetes mellitus 2 in (MGI:1355273), nicotine induced locomotor activity (MGI:3629978), skull morphology (MGI:2149704)
15	21	17	21	BMD	angiogenesis due to FGF2 QTL (MGI:3046557), dietary obesity (MGI:107856), femur geometry (MGI:3512951), liver Gapd decay rate (MGI:3521870)
15	27	19	33	Lc.V	alopecia areata (MGI:2677753), angiogenesis due to FGF2 QTL (MGI:3046557), accelerator of polyoma-induced mammary tumors (MGI:1891292), bronchial hyperresponsiveness (MGI:104734), bone mineral density (MGI:1349437), body weight QTL (MGI:3531525), cocaine related behavior (MGI:2151010), granulosa cell tumorigenesis (MGI:1330297), habituation (MGI:3707208), hepatic fibrogenesis (MGI:2178324), heart rate quantitative locus (MGI:2662842), IGF-1 serum levels (MGI:3046875), liver Gapd decay rate (MGI:3521871), pain (MGI:2384610), T cell receptor induced activation (MGI:1306783), vertebral morphology and mechanical traits (MGI:3697201), vertebral trabecular bone trait (MGI:3045059)
17	4	4	10	BMD	adiposity (MGI:3531543), obesity QTL (MGI:2686912), obesity QTL (MGI:1100507)

Table S5: A comprehensive list of all the previously identified murine QTL which overlap with the ones found in this study. The first five columns show the chromsome, peak position, and starting and ending positions of the 95% confidence interval for each QTL as determined in this study. The fifth column shows the overlapping QTL from the QTLArchive. The shaded rows indicate QTL which do not overlap at all with BMD and are of particular interest for further investigation.

Chr.	Pos.(cM)	Start	End	Phen.	Overlapping QTL (MGI Accession ID)
17	4	4	14	Ct.Th.R.sd	adiposity (MGI:3531543), femur geometry (MGI:3512953), mandible length (MGI:3528323), obesity QTL (MGI:2686912), obesity QTL (MGI:1100507), organ weight (MGI:3639982), small effect CIA locus (MGI:3717848), skull morphology (MGI:2149707), vertebral morphology and mechanical traits (MGI:3697202)
17	44	34	52	Lc.V	age related hearing loss (MGI:3514038), acute lung injury QTL (MGI:1345620), bone mechanical trait (MGI:3511312), bone mineral density (MGI:3694913), free fatty acid level (MGI:3036906)
18	16	2	16	Ct.Th.R.sd	heart failure modifier (MGI:3590238), insulin dependent diabetes susceptibility (MGI:3606917), modifier of Min (MGI:3697679), orthodenticle homolog 2 (Drosophila) modifier (MGI:2445326)
18	18	8	26	Lc.V	adiposity (MGI:2149049), angle of entrainment (MGI:2151088), bone mineral density (MGI:2389103), body weight, QTL (MGI:1890414), body weight at necropsy (MGI:3639985), heart failure modifier (MGI:3590238), immunoregulatory (MGI:3032863), kilocalorie (MGI:2151780), leishmaniasis resistance (MGI:2656546), lung tumor shape-determining (MGI:2664838), modifier of engrailed QTL (MGI:3715500), non-insulin-dependent diabetes mellitus (MGI:1277974), orthodenticle homolog 2 (Drosophila) modifier (MGI:2445326), susceptibility to lung cancer (MGI:1335086)
18	26	4	42	Ca.Dn	adiposity (MGI:2149049), angle of entrainment (MGI:2151088), bleomycin-induced pulmonary fibrosis (MGI:3606260), bone mineral density (MGI:2389103), body weight, QTL (MGI:1890414), body weight at necropsy (MGI:3639985), HDL level (MGI:3514363), heart failure modifier (MGI:2686941), heart failure modifier (MGI:3590238), insulin dependent diabetes susceptibility (MGI:3606916), insulin dependent diabetes susceptibility (MGI:3606917), immunoregulatory (MGI:3032863), kilocalorie (MGI:2151780), limb length QTL (MGI:3042424), leishmaniasis resistance (MGI:2656546), lung squamous cell carcinoma (MGI:3040191), lung tumor shape-determining (MGI:2664838), macronutrient intake, fat (MGI:2151778), modifier of engrailed QTL (MGI:3715500), non-insulin-dependent diabetes mellitus (MGI:1277974), ocular degeneration with sex reversal modifier (MGI:2653281), orthodenticle homolog 2 (Drosophila) modifier (MGI:2445326), susceptibility to lung cancer (MGI:1335086), vertebral trabecular bone trait (MGI:3045061)
18	40	18	48	BMD	adiposity (MGI:2149049), age-related hearing loss (MGI:3514195), angle of entrainment (MGI:2151088), bleomycin-induced pulmonary fibrosis (MGI:3606260), bone mineral density (MGI:2389103), body weight, QTL (MGI:1890414), body weight at necropsy (MGI:3639985), cocaine induced activation (MGI:3040205), HDL level (MGI:3514363), wound healing/regeneration (MGI:2669604), heart failure modifier (MGI:2686941), insulin dependent diabetes susceptibility (MGI:3606916), immunoregulatory (MGI:3032863), kilocalorie (MGI:2151780), limb length QTL (MGI:3042424), leishmaniasis resistance (MGI:2656546), lung squamous cell carcinoma (MGI:3040191), lung tumor shape-determining (MGI:2664838), macronutrient intake, fat (MGI:2151778), modifier of engrailed QTL (MGI:3715500), non-insulin-dependent diabetes mellitus (MGI:1277974), ocular degeneration with sex reversal modifier (MGI:2653281), pulmonary adenoma resistance (MGI:1855887), pulmonary adenoma susceptibility (MGI:108459), susceptibility to lung cancer (MGI:1335086), vertebral trabecular bone trait (MGI:3045061)

QTL Model Penalties

In table S6 the estimated 1 and 2 QTL Penalty values obtained from two loci scan with 1000 permutations are shown.

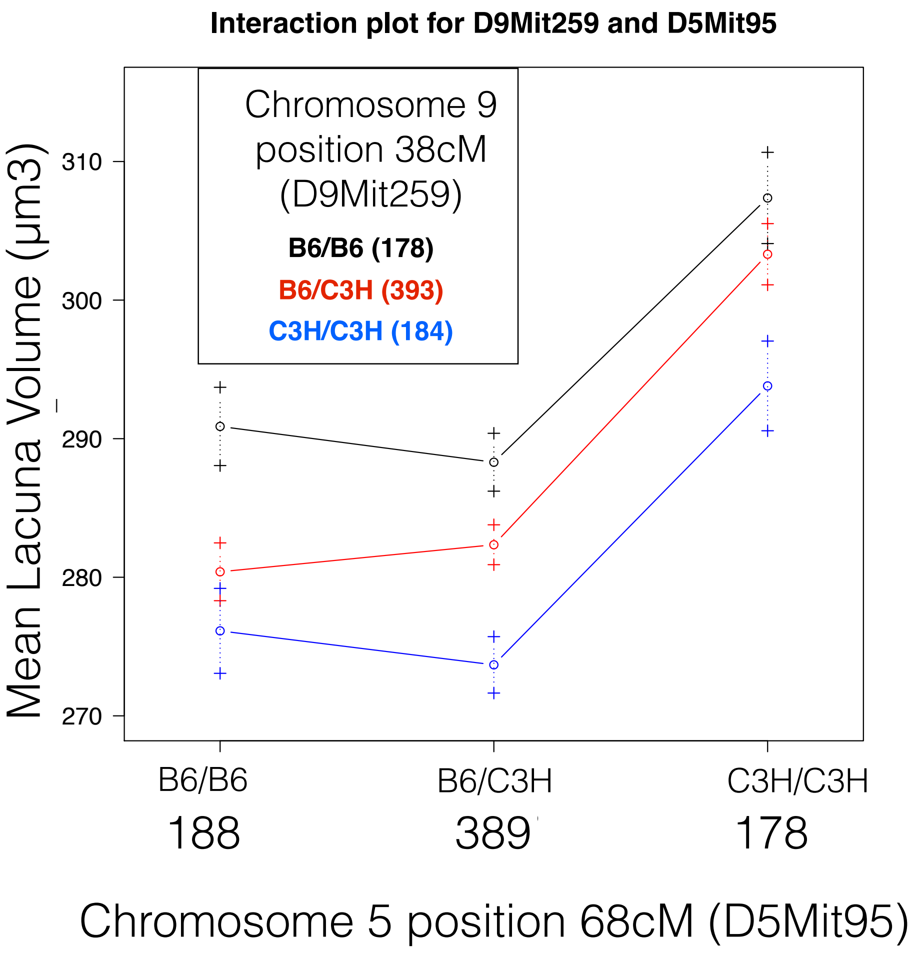

Figure S5 This shows two important markers close to the loci used in the Lacuna Volume model. The markers were selected for being strong contributors (>10 LOD, >4% explained variance) The first (D5Mit95) is plotted on the X-axis (animal count shown below the label) and shows a much larger phenotype value at the genotype C3H/C3H and a strong B6 dominance. The other marker (D9Mit259) shows the opposite effect where the B6/B6 genotype increases the phenotype value. They together contribute over 11% of the variance. Furthermore while the D9Mit259 marker has not yet been associated to any bone structural properties, the D5Mit95 has been associated to a mid-diaphysis elliptical semi-axis metric measured using BALBcJ x C57BL/6J mice. [35].

Phenotype	Main	Heavy	Light
BMD	2.810048	4.830833	2.701307
Radial Cortical Thickness	2.846717	5.108932	3.003231
Cortical Thickness Variation	2.716231	5.018889	2.955954
Canal Density	2.920043	5.102311	2.821378
Lacuna Density	2.920165	5.788332	3.566711
Lacuna Volume	2.947109	4.928250	2.789138
Lacuna Stretch	2.897525	6.241428	4.173224

Table S6 Estimated 1 and 2 QTL Penalty values (in LOD) obtained from two loci scan with 1000 permutations.

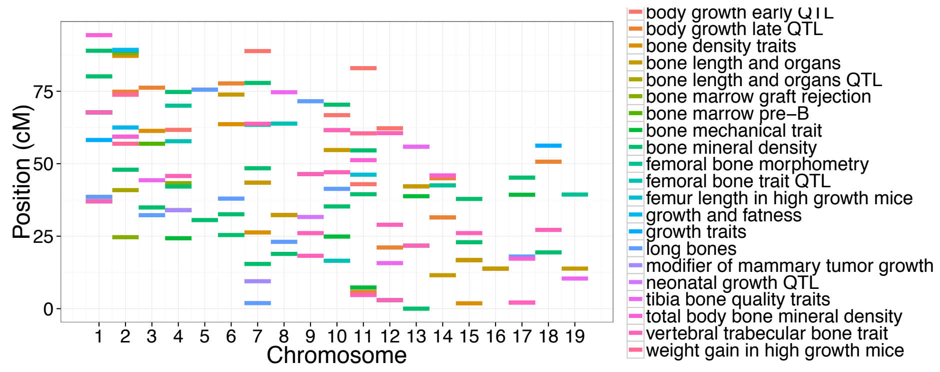

Figure S6 The graph shows the existing QTL obtained from the QTLArchive database matching the keywords bone or growth. The map provides a template for comparing the results of this study. The x-axis denotes the chromosome and the y-axis shows the position of the QTL within the chromosome. The color indicates the phenotype or name of the given QTL.

Homologous Human Genes

The QTL were compared with the available database of homologous human genes from the Mouse Genome Informatics Database (http://informatics.jax.org/, The Jackson Laboratory, Bar Harbor, USA). The database was selected for mouse - human homologies. From these results the ones which fell in the 95% confidence interval for the mouse QTL in this study were taken. The results are summarized in a plot in figure S7. Since the higher LOD regions are more relevant for homologues, figure S10 shows the LOD score against the homologous genes. To better portray the overlap between the QTL and homologous genes, we produced a second plot with the links between mouse phenotype, mouse genome, and human genome (fig. S9). The figure better shows the amount of localization for specific regions of the mouse genome to the human genome, but does not make the connection to single genes any clearer. While some regions (chromosome 1 and 11) are very strongly conserved mapping mostly to one region of the human genome, others like 5, 15, and 17 map almost uniformly strongly across to a number of different spots in the human genome.

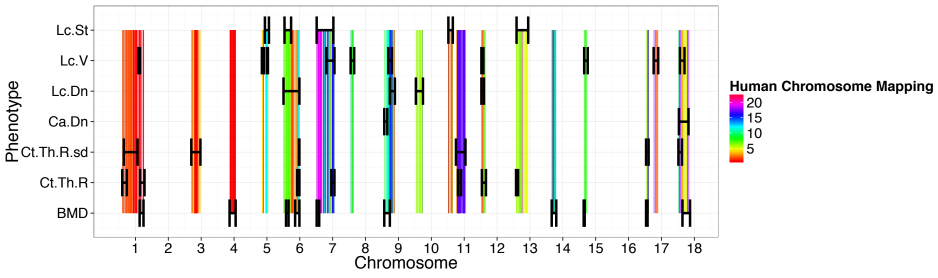

Figure S7 The chart shows the QTL identified for the various phenotypes identified in this study against chromosome position. The x-axis denotes chromosome position with chromosome number corresponding to the rounded integer value and position normalized to the ±0.5 range around this value. The range for the QTL indicates the 95% confidence interval based on LOD-score. The y-axis shows phenotype. The colored vertical lines indicate the mouse phenotype which is closest to given QTL.

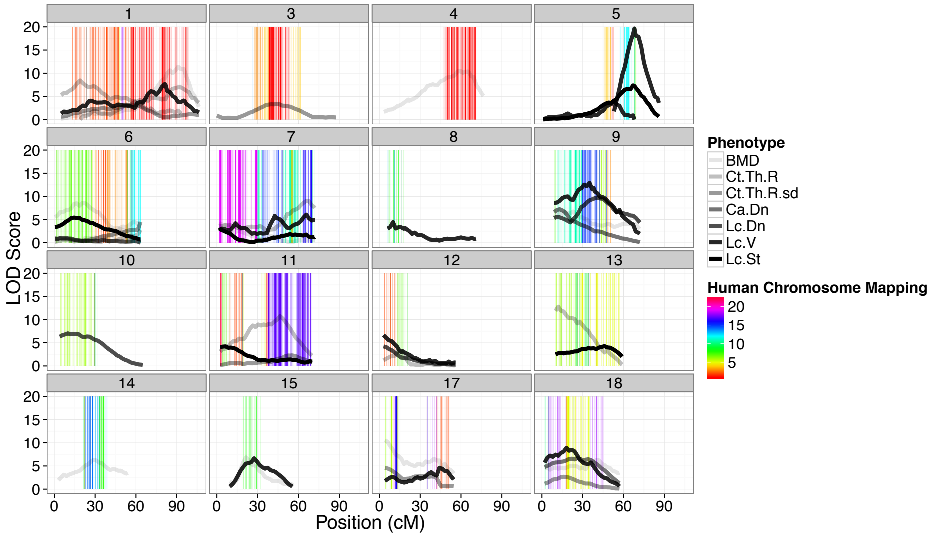

Figure S8 The graph shows the location of the mouse human-human gene homologues in context of the LOD score determined for the QTL models. Each chromosome is shown as a small box. The x-axis is the position along that specific chromosome and the y-axis is the LOD score. The colored vertical lines indicate the human chromosome to which the given mouse gene maps for the homologous genes.

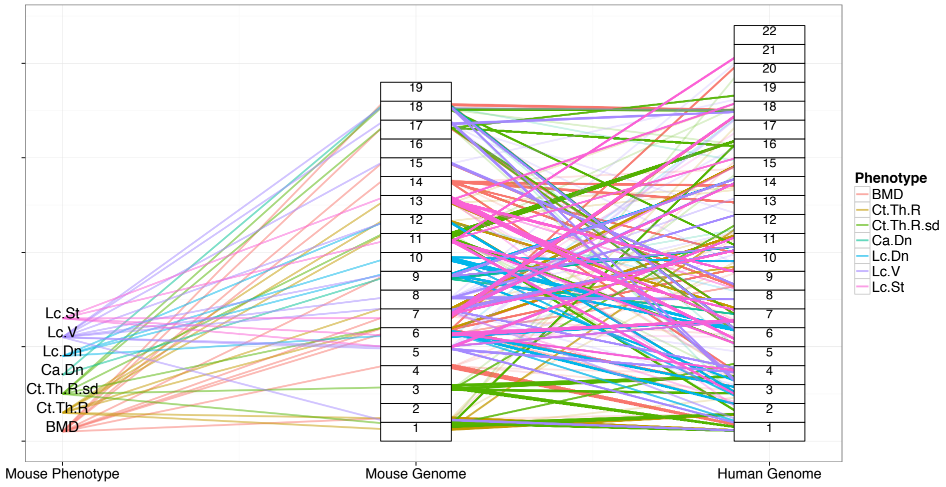

Figure S9 The graph shows the connection between phenotypes (right), the mouse genome (middle), and the human genome (right). The lines between the phenotypes and the mouse genome are the QTL identified in this paper. The lines between the mouse genome and human genome are homologous genes as found in the MGI database which overlap with the QTL.

Hardy Weinberg Equilibrium Test

We performed the HW Equilibrium test on the markers data (dropping missing or unknown genotypes) and

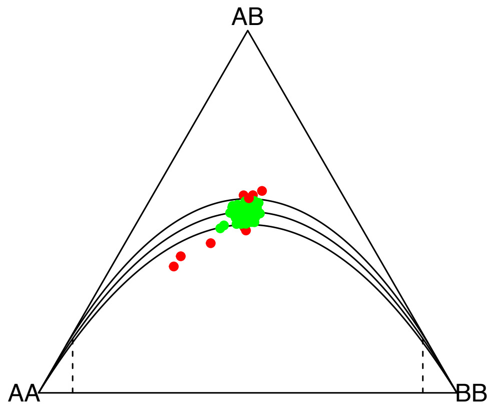

Figure S10 The figure shows the ternary plot for the Hardy Weinberg analysis of the genotypes distribution at each marker. Each marker is represented as a point, green is within the expected range and red are the points which fall out of this tolerance.

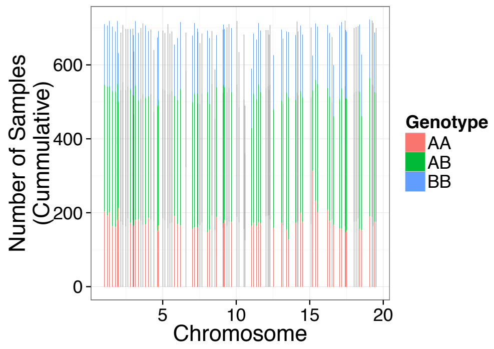

Figure S11 Sample count at each marker position (x-axis) for each genotype option (color), plotted in a stacked manner so the different genotypes are additive and the final height is the number of samples.

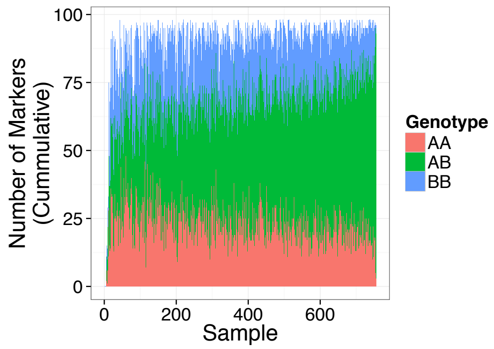

Figure S12 Sample number is shown as the position (x-axis) for each genotype option (color), plotted in a stacked manner so the different the height represents the number of markers at that position.

Normality of examined phenotypes

Strictly speaking a phenotype must be either normally distributed or binary to be fit to a QTL model. Several of the phenotypes examined, however, fail the normality test. We proceeded with the standard analysis for several reasons. First, while the distribution of one of the phenotypes appears to be bimodal, a significant amount of information is lost when a threshold is applied to convert the data to a binary distribution. For the others, a transformation of the variables (log, exp, etc.) would perhaps make the distribution closer to the desired normal, but take away the biological significance of the metric examined, and thus the contribution of various loci would no longer be directly applicable to that biological trait. Finally, since we are seeking to find potential areas of the genome and genes for further investigation in humans, a quantitative understanding of exactly what percentage of the metric comes from each locus is not important because it will in all likelihood not be transferable to humans.

References

1. Norris, F.C., Wong, M.D., Greene, N.D.E., Scambler, P.J., Weaver, T., Weninger, W.J., Mohun, T.J., Henkelman, R.M., Lythgoe, M.F.: A coming of age: advanced imaging technologies for characterising the developing mouse. Trends in Genetics **29**(12), 700–711
2. de Souza, N.: High-throughput phenotyping. Nature Methods **7**(1), 36–36 (2010). doi:10.1038/nmeth.f.289
3. Pardo-Martin, C., Allalou, A., Medina, J., Eimon, P.M., Wählby, C., Fatih Yanik, M.: High-throughput hyperdimensional vertebrate phenotyping. Nature Communications **4**, 1467 (2013). doi:10.1038/ncomms2475
4. Jansen, R.C., Stam, P.: High resolution of quantitative traits into multiple loci via interval mapping. Genetics **136**(4), 1447–55 (1994)
5. Topp, C.N., Iyer-Pascuzzi, A.S., Anderson, J.T., Lee, C.-R., Zurek, P.R., Symonova, O., Zheng, Y., Bucksch, A., Mileyko, Y., Galkovskyi, T., Moore, B.T., Harer, J., Edelsbrunner, H., Mitchell-Olds, T., Weitz, J.S., Benfey, P.N.: 3D phenotyping and quantitative trait locus mapping identify core regions of the rice genome controlling root architecture. Proceedings of the National Academy of Sciences of the United States of America **110**(18), 1695–704 (2013). doi:10.1073/pnas.1304354110
6. Raman, P., Raman, R., Newman, B., Venkatraman, R., Raman, B., Robinson, T.E.: Development and validation of automated 2D-3D bronchial airway matching to track changes in regional bronchial morphology using serial low-dose chest CT scans in children with chronic lung disease. Journal of digital imaging **23**(6), 744–54 (2010). doi:10.1007/s10278-009-9199-3
7. Huang, X., Feng, Q., Qian, Q., Zhao, Q., Wang, L., Wang, A., Guan, J., Fan, D., Weng, Q., Huang, T., Dong, G., Sang, T., Han, B.: High-throughput genotyping by whole-genome resequencing. Genome research **19**(6), 1068–76 (2009). doi:10.1101/gr.089516.108
8. Wang, Z., Clavijo, C.A., Roessl, E., van Stevendaal, U., Koehler, T., Hauser, N., Stampanoni, M.: Image fusion scheme for differential phase contrast mammography. Journal of Instrumentation **8**(07), 07011–07011 (2013). doi:10.1088/1748-0221/8/07/C07011
9. Hellrung, D.J., Rossi, G., Link, C.J.: High-throughput fluorescent screening of transgenic animals: phenotyping and haplotyping. Cytometry. Part A : the journal of the International Society for Analytical Cytology **69**(10), 1092–5 (2006). doi:10.1002/cyto.a.20328
10. Tanabata, T., Shibaya, T., Hori, K., Ebana, K., Yano, M.: SmartGrain: high-throughput phenotyping software for measuring seed shape through image analysis. Plant physiology **160**(4), 1871–80 (2012). doi:10.1104/pp.112.205120
11. Team, R.C.: R: A Language and Environment for Statistical Computing. R Foundation for Statistical Computing, Vienna, Austria (2013)
12. Jiao, F., Chiu, H., Jiao, Y., de Rijk, W.G., Li, X., Eckstein, E.C., Beamer, W.G., Gu, W.: Quantitative trait loci for tibial bone strength in C57BL/6J and C3H/HeJ inbred strains of mice. Journal of genetics **89**(1), 21–7 (2010)
13. Jiao, Y., Chiu, H., Fan, Z., Jiao, F., Eckstein, E.C., Beamer, W.G., Gu, W.: Quantitative trait loci that determine mouse tibial nanoindentation properties in an F2 population derived from C57BL/6J x C3H/HeJ. Calcified tissue international **80**(6), 383–90 (2007). doi:10.1007/s00223-007-9030-4
14. Ruffoni, D., Kohler, T., Voide, R., Wirth, A.J., Donahue, L.R., Müller, R., van Lenthe, G.H.: High-throughput quantification of the mechanical competence of murine femora - A highly automated approach for large-scale genetic studies. Bone **55**(1), 216–21 (2013). doi:10.1016/j.bone.2013.02.015
15. Mader, K., Marone, F., Mikuljan, G., Isenegger, A., Stampanoni, M.: High-throughput, fully-automatic, synchrotron-based microscopy station at TOMCAT. Journal of Synchrotron Radiation **18**(2), 117–124 (2011). doi:10.1107/S0909049510047370
16. Blomfeldt, R., Törnkvist, H., Ponzer, S., Söderqvist, A., Tidermark, J.: Internal fixation versus hemiarthroplasty for displaced fractures of the femoral neck in elderly patients with severe cognitive impairment. The Journal of bone and joint surgery. British volume **87**(4), 523–9 (2005). doi:10.1302/0301-620X.87B4.15764

17. Snyder SM, S.E., Snyder, S.M., Schneider, E.: Estimation of mechanical properties of cortical bone by computed tomography. J Orth Res **9**(3), 422–431 (1991). doi:10.1002/jor.1100090315
18. Beamer, W.G., Shultz, K.L., Coombs, H.F., Horton, L.G., Donahue, L.R., Rosen, C.J.: Multiple quantitative trait loci for cortical and trabecular bone regulation map to mid-distal mouse chromosome 4 that shares linkage homology to human chromosome 1p36. Journal of bone and mineral research : the official journal of the American Society for Bone and Mineral Research (2011). doi:10.1002/jbmr.515
19. Bouxsein, M.L., Uchiyama, T., Rosen, C.J., Shultz, K.L., Donahue, L.R., Turner, C.H., Sen, S., Churchill, G.A., Müller, R., Beamer, W.G.: Mapping quantitative trait loci for vertebral trabecular bone volume fraction and microarchitecture in mice. Journal of bone and mineral research : the official journal of the American Society for Bone and Mineral Research **19**(4), 587–99 (2004). doi:10.1359/JBMR.0301255
20. Kohler, T., Stauber, M., Donahue, L.R., Müller, R., Rae, L.: Automated compartmental analysis for high-throughput skeletal phenotyping in femora of genetic mouse models. Bone **41**(4), 659–67 (2007). doi:10.1016/j.bone.2007.05.018
21. Koller, D.L.: Genome Screen for QTLs Contributing to Normal Variation in Bone Mineral Density and Osteoporosis. Journal of Clinical Endocrinology & Metabolism **85**(9), 3116–3120 (2000). doi:10.1210/jc.85.9.3116
22. Rosen, C.J., Beamer, W.G., Donahue, L.R.: Defining the genetics of osteoporosis: using the mouse to understand man. Osteoporosis international : a journal established as result of cooperation between the European Foundation for Osteoporosis and the National Osteoporosis Foundation of the USA **12**(10), 803–10 (2001)
23. Elefteriou, F., Yang, X.: Genetic mouse models for bone studies—Strengths and limitations. Bone **49**(6), 1242–1254 (2011). doi:10.1016/j.bone.2011.08.021
24. Mader, K.S., Schneider, P., Müller, R., Stampanoni, M.: A quantitative framework for the 3D characterization of the osteocyte lacunar system. Bone **57**(1), 142–154 (2013). doi:10.1016/j.bone.2013.06.026
25. Volkman, S.K., Galecki, A.T., Burke, D.T., Miller, R.A., Goldstein, S.A.: Quantitative trait loci that modulate femoral mechanical properties in a genetically heterogeneous mouse population. Journal of bone and mineral research : the official journal of the American Society for Bone and Mineral Research **19**(9), 1497–505 (2004). doi:10.1359/JBMR.040506
26. Devoto, M., Falchi, M.: Genetic mapping of quantitative trait loci for disease-related phenotypes. Methods in molecular biology (Clifton, N.J.) **871**, 281–311 (2012). doi:10.1007/978-1-61779-785-9_15
27. Schneider, P., Stauber, M., Voide, R., Stampanoni, M., Donahue, L.R., Müller, R.: Ultrastructural Properties in Cortical Bone Vary Greatly in Two Inbred Strains of Mice as Assessed by Synchrotron Light Based Micro- and Nano-CT. Journal for Bone Mineral Research **22**(10), 1557–1570 (2007). doi:10.1359/JBMR.070703
28. Hildebrand, T., Ruegsegger, P.: A new method for the model-independent assessment of thickness in three-dimensional images. Journal of Microscopy **185**(1), 67–75 (1997). doi:10.1046/j.1365-2818.1997.1340694.x
29. Broman, K.W., Wu, H., Sen, S., Churchill, G.A.: R/qtl: QTL mapping in experimental crosses. Bioinformatics (Oxford, England) **19**(7), 889–90 (2003)
30. Broman, K.W., Sen, S.: A Guide to QTL Mapping with R/qtl. Springer (2009). http://www.springer.com/life+sciences/systems+biology+and+bioinformatics/book/978-0-387-92124-2http://books.google.com/books?hl=en&lr=&id=tPoXT_dCguQC&oi=fnd&pg=PA1&dq=A+Guide+to+QTL+Mapping+with+R/qtl&ots=8tscXutQz9&sig=T2EQ2sI3bL8GxWuc1rxSFmHkKeI Accessed 26/10/11
31. Griffiths, A.J., Miller, J.H., Suzuki, D.T., Lewontin, R.C., Gelbart, W.M.: An Introduction to Genetic Analysis. W. H. Freeman, New York (2000). http://www.ncbi.nlm.nih.gov/books/NBK21766/
32. Arends, D., Prins, P., Jansen, R.C., Broman, K.W.: R/qtl: high-throughput multiple QTL mapping. Bioinformatics (Oxford, England) **26**(23), 2990–2 (2010). doi:10.1093/bioinformatics/btq565
33. Wickham, H.: Ggplot2: Elegant Graphics for Data Analysis. Springer, ??? (2009). http://had.co.nz/ggplot2/book
34. Wickham, H.: The Split-Apply-Combine Strategy for Data Analysis. Journal of Statistical Software **40**(1), 1–29 (2011)
35. Volkman, S.K., Galecki, A.T., Burke, D.T., Paczas, M.R., Moalli, M.R., Miller, R.A., Goldstein, S.A.: Quantitative trait loci for femoral size and shape in a genetically heterogeneous mouse population. Journal of bone and mineral research : the official journal of the American Society for Bone and Mineral Research **18**(8), 1497–505 (2003). doi:10.1359/jbmr.2003.18.8.1497
